# Effects of Multilevel and Multidomain Interventions on Glycemic Control in U.S. Hispanic Populations

**DOI:** 10.3390/ijerph22091345

**Published:** 2025-08-28

**Authors:** Laura Bianco, Sofía I. Uranga, Alexander W. Rodriguez, Raj Shetty, Erin M. Staab, Melissa I. Franco-Galicia, Amber N. Deckard, Nikita C. Thomas, Wen Wan, Jason T. Alexander, Arshiya A. Baig, Neda Laiteerapong

**Affiliations:** 1 Weinberg College of Arts and Sciences, Northwestern University, Evanston, IL 60208, USA; 2 Section of General Internal Medicine, Department of Medicine, University of Chicago, Chicago, IL 60637, USA

**Keywords:** type 2 diabetes, HbA1c, Hispanic, Latino, Latina, Latinx, ethnicity

## Abstract

Hispanic populations in the U.S. have a high prevalence of type 2 diabetes and its complications. It has been proposed that interventions targeting multiple levels and domains of influence are needed to address health disparities, but more evidence is needed regarding the most effective approaches. We aimed to review the effects of non-pharmacological interventions on glycemic control among Hispanic persons with diabetes, overall and by level and domain of intervention. A systematic review (PubMed, Scopus, PsycInfo, CINAHL; 1985–2019) identified randomized trials reporting HbA1c outcomes for Hispanic populations. Article review, data extraction, and risk of bias assessment were completed by independent reviewers. Level and domain of intervention were assigned based on the National Institute on Minority Health and Health Disparities Research Framework. Random-effects meta-analyses estimated pooled effect sizes. Quality of evidence was rated based on the GRADE framework. Forty-eight trials met inclusion criteria, representing various Hispanic populations (*n* = 18 Mexican, *n* = 5 Puerto Rican, *n* = 1 Dominican, *n* = 4 multiple, *n* = 20 unspecified) and enrolling 9185 total participants. Overall, interventions decreased HbA1c by −0.32% (95% CI: −0.44% to −0.20%, I^2^ = 68%, strength of evidence: moderate). Multi-level, multi-domain interventions decreased HbA1c by −0.41% (−0.61% to −0.21%, I^2^ = 74%, strength of evidence: moderate). Few interventions addressed community (*n* = 3), society (*n* = 0), or physical/built environment (*n* = 1). Non-pharmacological interventions have modestly decreased HbA1c among Hispanic persons with diabetes. Multi-level, multi-domain interventions are promising, but more research is needed on interventions that target social and environmental structures.

## 1. Introduction

Hispanic populations are the largest and one of the fastest-growing minority groups in the U.S., accounting for >19% of the population [1,2]. Hispanic populations have a prevalence of type 2 diabetes that exceeds the national average [1]. Hispanic adults in the U.S. are 70% more likely to be diagnosed with type 2 diabetes than non-Hispanic White adults [3]. Furthermore, Hispanic persons face increased comorbidities and complications associated with diabetes than their non-Hispanic White counterparts, including higher rates of hypertension and diabetic retinopathy [4]. These disparities are due to multiple factors that exert influence at individual, interpersonal, community, and societal levels [5].

Evidence is needed on the most effective types of interventions to control diabetes and address disparities. Diabetes self-management education (DSME) has been shown to modestly reduce hemoglobin A1c (HbA1c) in Hispanic populations. Prior meta-analyses have found the largest effects for DSME interventions that were culturally tailored, team-led (vs. solo provider), and delivered to individual patients (vs. groups) [6,7]. Other reviews, however, have concluded that the most successful DSME interventions included group education and support [8,9].

One systematic review and meta-analysis of interventions that utilized telehealth for Black and Hispanic patients aimed to compare effects by level of intervention, but the authors identified only two studies that addressed any level beyond the individual patient [10]. No reviews have categorized and analyzed interventions per the National Institute on Minority Health and Health Disparities (NIMHD) research framework, which outlines multiple levels (individual, interpersonal, community, societal) and domains (behavioral, physical/built environment, sociocultural, health care system) that interventions can target to affect health outcomes [11].

The purpose of this study was to review randomized controlled trials of non-pharmacological interventions focused on Hispanic persons with diabetes in the U.S. and conduct a meta-analysis to evaluate their effectiveness at lowering HbA1c, overall and by level and domain of intervention, per the NIMHD research framework.

## 2. Materials and Methods

This review was registered with the International Prospective Register of Systematic Reviews (PROSPERO, CRD42019122625) and conformed to the Preferred Reporting Items for Systematic Reviews and Meta-Analyses (PRISMA) standards (Table S1: PRISMA Checklist). From this review of the literature, we previously published a meta-analysis of DSME interventions across racial/minority ethnic groups [12]. In this paper, we explore interventions within Hispanic populations specifically.

### 2.1. Search Strategy

As described in our previous report, a literature search was conducted of English-language articles published from 1985 to 2019 using PubMed, Scopus, PsycInfo, and CINAHL databases. Various search terms were used, all structured around diabetes, study design, language, race/ethnicity, and disparities (Table S2: Search Terms). Duplicates were removed, and articles underwent a title and abstract review with each article screened by two team members. If either person thought the title and abstract did not clearly meet criteria for exclusion, the article was moved forward for full paper review. A team of nine, including six of the authors and three trained research assistants, reviewed full papers. Each paper was independently assessed by two team members, with questions and discrepancies discussed to consensus during regular team meetings.

### 2.2. Inclusion and Exclusion Criteria

We included studies of non-pharmacological interventions for adults with type 2 diabetes that reported HbA1c results and enrolled a majority Hispanic population (at least 50% Hispanic). Studies were also included if they had less than 50% Hispanic individuals but reported HbA1c results stratified by ethnicity. Furthermore, studies were only included if the study design was a randomized controlled trial and the study duration was at least 3 months (Table S3: Inclusion Criteria According to PICOS Framework).

### 2.3. Data Extraction

Two independent reviewers collected data (study, intervention, and participant characteristics; HbA1c results) from each article, and discrepancies were checked by a third reviewer. Interventions were categorized by levels and domains of influence as outlined in the NIMHD Research Framework. Levels of influence include individual, interpersonal, community, and societal. Domains of influence include behavioral, physical/built environment, sociocultural environment, and health care system. Level and domain were coded by two independent reviewers, with discrepancies settled by a third reviewer or discussion with the research team.

### 2.4. Quality Review

The Cochrane Risk of Bias for randomized trials tool was used to assess the risk of bias for each study (Cochrane Handbook for Systematic Reviews of Interventions, 2021, [13]) with independent ratings from two reviewers entered into individual REDCap forms. Discrepancies in the rating forms were identified and discussed to consensus with the principal investigator or senior project staff. Overall quality of evidence was determined according to the GRADE (Grading of Recommendations, Assessment, Development, and Evaluations) framework [14].

### 2.5. Statistical Analysis

We conducted meta-analysis using the DerSimonian–Laird random-effects model and calculated a weighted average of the estimated effects within individual studies (a pooled effect estimate). Weights were determined based on the inverse of the within-study variance and between-studies variance for each study. We then constructed a z-test and its 95% confidence interval (CI) of pooled intervention effects [15]. Heterogeneity was assessed with the I^2^ statistic. We conducted sub-group analyses by type of intervention and preferred language of participants. We constructed funnel plots and conducted Egger’s test to assess publication bias.

## 3. Results

**Figure 1:**
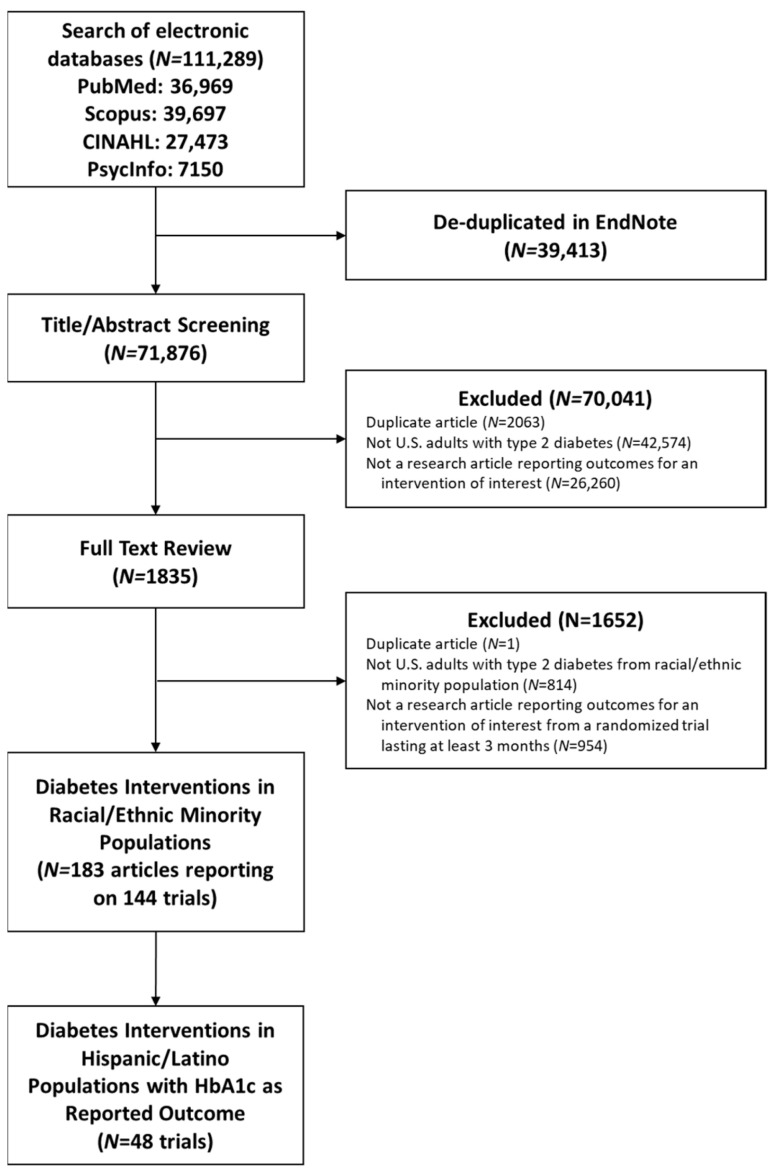
PRISMA flow diagram for systematic review of non-pharmacological intervention trials in U.S. Hispanic populations with type 2 diabetes.

The initial database searches yielded 111,289 articles. Duplicates were removed, and article titles and abstracts were screened for eligibility, after which 1835 underwent full text review (Figure 1). Ultimately, 48 trials met criteria and were included in the meta-analysis. The trials took place across the U.S., with sixteen in the West, fifteen in the Northeast, twelve in the South, and seven in the Midwest; one study was conducted in multiple regions. Average study duration was 6.5 months (range 3–12 months) (Table 1).

The trials enrolled a total of 9185 participants, the majority of whom were female with average age ranging from 45 to 70. More than a third of trials (*n* = 18) primarily enrolled Mexican/Mexican American participants. Eight trials primarily enrolled Caribbean/Caribbean American participants (*n* = 5 Puerto Rican, *n* = 1 Dominican, *n* = 2 multiple Caribbean origins). The remaining trials enrolled participants from various backgrounds (*n* = 2) or did not specify the origins of Hispanic participants (*n* = 20) (Supplemental Table S4: Characteristics of Participants in Trials of Non-Pharmacological Interventions Among U.S. Hispanic Populations with Type 2 Diabetes, 1985–2019).

Nearly all interventions (*n* = 45) included some form of diabetes self-management education. Interventions were often delivered by promotoras, community health workers, or lay health leaders (*n* = 17); nurses, health educators, or nutritionists (*n* = 14); or a combination of these roles (*n* = 5). A small number of interventions were delivered by medical assistants (*n* = 2), therapists (*n* = 2), pharmacists (*n* = 1), or physicians (*n* = 1) or were entirely technology-based (e.g., automated text messaging program) (*n* = 4).

Half of the interventions were multi-level (*n* = 25), and three-quarters (*n* = 36) were multi-domain; twenty-two were both multi-level and multi-domain. All but one intervention addressed the individual level, twenty-five addressed the interpersonal level, three addressed the community level, and none addressed the societal level. All but two interventions targeted the behavioral domain, thirty-two targeted the sociocultural environment, thirteen targeted the health care system, and one targeted the physical/built environment. The most commonly targeted level/domain combination was the individual level with the behavioral and sociocultural domains (*n* = 13), followed by the individual and interpersonal levels with the behavioral and sociocultural domains (*n* = 10).

Overall, compared to control groups, interventions decreased HbA1c by −0.32% (95% CI: −0.44% to −0.20%, I^2^ = 68%) (Table 2, Figure S1: Forest Plots). Results were similar when we limited analyses to studies in which >90% of participants were Hispanic (*n* = 35), with a reduction in HbA1c of −0.37% (−0.51% to −0.23%, I^2^ = 66%, *n* = 35). In trials where the majority of participants’ preferred language was Spanish, HbA1c decreased by −0.34% (−0.47% to −0.21%, I^2^ = 51%). 

Multi-level interventions decreased HbA1c by −0.39% (−0.57% to −0.21%, I^2^ = 73%); single-level interventions decreased HbA1c by −0.25% (−0.40% to −0.10%, I^2^ = 50%). Multi-domain interventions decreased HbA1c by −0.31% (−0.46% to −0.16%, I^2^ = 74%); single-domain interventions decreased HbA1c by −0.32% (−0.46% to −0.1%, I^2^ = 0%). Interventions that were both multi-level and multi-domain decreased HbA1c by −0.41% (−0.61% to −0.21%, I^2^ = 74%).

In sub-analyses by specific level/domain combination, interventions that targeted the individual and interpersonal levels and the behavioral and sociocultural domains showed the largest decrease in HbA1c (−0.54%; −0.89% to −0.19%, I^2^ = 75%), followed by interventions that targeted the individual and interpersonal levels and the behavioral, sociocultural, and health care domains (−0.46%; −0.85% to −0.08%, I^2^ = 68%).

Quality review identified twenty-six trials with low risk of bias, nineteen with some concerns of bias, and three with high risk of bias (Table S5: Risk of Bias in Randomized Controlled Trials of Non-Pharmacological Interventions Among U.S. Hispanic Populations with Type 2 Diabetes, 1985–2019). Concerns about bias fell within the domains of deviations from the intended intervention and missing outcome data. There were no concerns related to randomization, measurement, or selection of reported results. Funnel plot asymmetry and Egger’s test (*p* = 0.03) indicated possible publication bias (Figure S2: Funnel Plots), and as a result, the strength of evidence was downgraded to moderate for our overall results (Table S6: GRADE Strength of Evidence Assessment for Non-Pharmacological Interventions Among U.S. Hispanic Populations with Type 2 Diabetes). Strength of evidence for multi-level interventions was rated moderate due to indirectness of the included studies, which focused largely on individual and interpersonal levels. Strength of evidence for multi-domain interventions rated was low due to inconsistency in the direction of results and concerns about bias related to missing data and deviations from intended interventions.

## 4. Discussion

This systematic review and meta-analysis investigated the effectiveness of non-pharmacological interventions on glycemic outcomes in U.S. Hispanic populations with type 2 diabetes. We identified 48 trials and found that interventions led to a HbA1c decrease (−0.32%) compared to control groups. Results were similar in trials enrolling primarily Spanish-speaking individuals (−0.34%). Multi-level, multi-domain interventions had a larger, though not statistically different, effect on HbA1c (−0.41%) than interventions overall. These effect sizes are modest, as a clinically important reduction in HbA1c is generally considered to be −0.5%.

Our results align with meta-analyses of DSME in Hispanic populations, which have reported an HbA1c reduction of about −0.25% overall and −0.42% for culturally tailored DSME [6,7]. In addition, we found that multi-level, multi-domain interventions showed promising results for Hispanic populations, such as interventions targeting individual and interpersonal levels plus behavioral and sociocultural domains (with some of these interventions also including the health care domain). According to the biopsychosocial model, a patient’s community, environment, and biological factors work together to shape their health. Therefore, addressing multiple aspects of a patient’s life may be integral to designing effective diabetes interventions [5].

Cultural influences play a central role in the health of minority populations in the U.S [64]. Altering interventions to account for the differences in culture can be key to overcoming barriers to a healthier lifestyle in Hispanic populations [65]. Indeed, previous research supports the importance of incorporating sociocultural context into diabetes interventions for Hispanic individuals, such as preferred foods and cultural values. For example, family plays a significant role in Hispanic cultures, and incorporating family involvement in interventions has been found to be a key component in increasing intervention adherence and efficacy [66]. Additionally, culturally responsive diet interventions, aligned with community preferences and fare, have improved weight control [67].

Our search yielded very few randomized controlled trials with interventions targeting the physical/built environment, the community level, or the societal level. Interventions targeting these areas might be scarcer because enacting structural changes to address social determinants (e.g., access to healthy food, green spaces, and culturally and linguistically appropriate health care) is more complex than targeting individual behaviors or knowledge. Additionally, these types of interventions might be harder to implement within a randomized controlled trial framework. Various interventions targeting these levels and domains have been developed, such as utilizing food banks to facilitate the distribution of healthy foods in low-income neighborhoods [68]. Future systematic reviews and meta-analyses could advance the field by assessing evidence from rigorous non-randomized evaluations of environmental, community, and societal interventions.

There are several limitations to be considered. We only examined HbA1c, and the interventions may have affected other important outcomes. We did not collect data on intervention fidelity or patient engagement which may impact the effectiveness of interventions. Classification of interventions’ level and domain of influence was subjective despite the rigorous independent coding process. We included only randomized controlled trials; HbA1c effects and levels/domains targeted may not be comparable in other types of studies. Our review included articles published through 2019; future work should assess studies from recent years.

As with all meta-analyses, the data used for analysis come from published works, and, therefore, results may not generalize to populations who are underrepresented in research on non-pharmacological diabetes interventions. The majority of participants in these studies were women. Very few studies reported having participants of South or Central American heritage; the majority targeted Mexican or Mexican American individuals or did not specify the origin of their participants. There is a high degree of heterogeneity across Hispanic populations and conclusions cannot be drawn from this work regarding the efficacy of interventions for different groups [5]. Additionally, generalizability of the results is limited to Hispanic populations in the U.S. because acculturation is known to influence the development of diabetes [64].

## 5. Conclusions

Hispanic persons continue to have increased diabetes incidence and burden compared to their White counterparts in the U.S. Thus, it is essential to investigate new and more effective interventions to treat this population. Future interventions should work to address additional domains (physical/built environment) and levels of influence (community and society). Given differences in the quality of care received by Hispanic individuals with diabetes, such as less frequent HbA1c testing, more effective interventions addressing the health care system domain might also be necessary [69,70]. Additionally, future research should investigate the effect of non-pharmacological interventions on other important diabetes-related outcomes, such as blood pressure and cognitive function, in Hispanic patients.

## Figures and Tables

**Table 1:** Randomized controlled trials of non-pharmacological interventions among U.S. Hispanic populations with type 2 diabetes, 1985–2019.

Study	Location	Enrolled in Trial/Screened for Eligibility, N	Follow-Up, Months	Control Arm	Intervention Arm	Level	Domain
**Individual/Behavioral**							
**Aponte 2017, [16]**	South Bronx, NY, USA	180/236	6	Usual care	Diabetes education including group sessions, home visits, and phone calls, delivered by a CHW	individual	behavioral
**Castaneda 2002, [17]**	Boston, MA, USA	62/100	4	Usual care	Supervised high-intensity progressive resistance training	individual	behavioral
**Chamany 2015, [18]**	South Bronx, NY, USA	941/9389	12	Printed self-management educational materials	Educational materials plus self-management support provided via phone by diabetes educators	individual	behavioral
**Frosch 2011, [19]**	Los Angeles, CA, USA	201/2438	6	Printed self-management educational materials	Self-management education video with a workbook and telephone coaching by a diabetes nurse educator	individual	behavioral
**Moncrieft 2016, [20]**	Miami, FL, USA	111/340	6	Usual care	Lifestyle intervention plus cognitive behavioral and social learning approaches to address depressive symptoms with individual and group sessions led by therapists	individual	behavioral
**Noël 1998, [21]**	Southwest TX, USA	596/NR	6	Assigned to either standard diabetes education curriculum or a nutritional focused program	Given the choice between standard diabetes education curriculum or nutrition focused program	individual	behavioral
**Palmas 2014, [22]**	New York, NY, USA	360/836	12	Usual care, educational materials, and quarterly phone calls	Diabetes education including group sessions, home visits, and phone calls, delivered by a CHW	individual	behavioral
**Sugiyama 2015, [23]**	Los Angeles, CA, USA	516/NR	6	Lectures on unrelated geriatrics topic	Group diabetes self-care sessions led by health educator	individual	behavioral
**Individual/Behavioral + Sociocultural**							
**Carrasquillo 2017, [24]**	Miami-Dade County, FL, USA	300/863	12	Usual care and mailed diabetes education materials	Home visits, phone calls, coaching, patient navigation, culturally relevant education, and group exercise sessions, delivered by CHW	individual	behavioral, sociocultural
**Castejón 2013, [25]**	Broward County, FL, USA	84/NR	3	Usual care	Culturally tailored pharmacist counseling on medication, nutrition, exercise, and self-care	individual	behavioral, sociocultural
**Fortmann 2017, [26]**	San Diego and Riverside Counties, CA, USA	126/825	6	Usual care	Culturally tailored text messaging program providing education, motivation, and support	individual	behavioral, sociocultural
**Gerber 2005, [27]**	Chicago, IL, USA	244/313	12	Multiple-choice diabetes quizzes delivered via computer kiosk in clinic waiting room	Diabetes education program delivered via computer kiosk in clinic waiting room, including culturally tailored lessons and patient testimonials	individual	behavioral, sociocultural
**Heisler 2014, [28]**	Detroit, MI, USA	188/391	3	Educational materials reviewed with CHW	Community-informed, personally tailored, interactive computerized diabetes medication decision aid reviewed with CHW	individual	behavioral, sociocultural
**Khanna 2014, [29]**	Oakland, CA, USA	75/78	3	Waitlist	Automated, interactive phone calls providing culture-concordant education and feedback on diet	individual	behavioral, sociocultural
**Lujan 2007, [30]**	Southwest TX, USA	150/160	6	Usual care	Culturally specific group classes led by promotoras and telephone follow-up	individual	behavioral, sociocultural
**Osborn 2010, [31]**	CT, USA	118/NR	3	Usual care	Brief, culturally tailored self-management intervention delivered by medical assistant	individual	behavioral, sociocultural
**Prezio 2013, [32]**	Dallas, TX, USA	180/1800	12	Waitlist	Culturally adapted diabetes management and education program led by CHW	individual	behavioral, sociocultural
**Rosal 2011, [33]**	MA, USA	252/1176	4	Usual care	Culturally tailored diabetes self-management program led by health educator, nutritionist, and/or lay leader	individual	behavioral, sociocultural
**Rothschild 2014, [34]**	Chicago, IL, USA	144/NR	12	Usual care and mailed education materials	Culturally appropriate self-management education delivered via CHW home visits	individual	behavioral, sociocultural
**Sixta 2008, [35]**	Webb County, TX, USA	131/761	6	Waitlist	Promotores-led culturally sensitive diabetes self-management course	individual	behavioral, sociocultural
**Wagner 2016, [36]**	Hartford, CT, USA	107/NR	3	Single group diabetes education session	Culturally sensitive, manualized group sessions led by CHW focused on relaxation techniques, stress management, and psychoeducation	individual	behavioral, sociocultural
**Individual + Interpersonal/Behavioral**							
**Ayala 2015, [37]**	Imperial County, CA, USA	336/1202	6	Usual care	Peer support intervention	individual, interpersonal	behavioral
**Burner 2018, [38]**	Los Angeles, CA, USA	44/745	3	Text message education program	Text message education program for both individual with diabetes and a designated support person	individual, interpersonal	behavioral
**Lorig 2008, [39]**	San Francisco Bay Area, CA, USA	533/765	6	Peer-led self-management program	Peer-led self-management program plus automated telephone reinforcement	individual, interpersonal	behavioral
**Individual + Interpersonal/Behavioral + Sociocultural**							
**Brown 2002, [40]**	Starr County, TX, USA	256/NR	6	Waitlist	Culturally tailored diabetes self-management education and group support sessions led by nurses, dietitians, and CHWs	individual, interpersonal	behavioral, sociocultural
**Brown 2005, [41]**	Starr County, TX, USA	216/NR	3	Compressed version of diabetes self-management education and support	Extended version of diabetes self-management education and support program	individual, interpersonal	behavioral, sociocultural
**Brown 2011, [42]**	Starr County, TX, USA	83/NR	6	Diabetes self-management education and support	Diabetes self-management education and support plus nurse case management	individual, interpersonal	behavioral, sociocultural
**McEwen 2017, [43]**	AZ, USA	157/929	3	Waitlist	Family-based, culturally tailored education and social support group sessions, home visits, and telephone calls delivered by nurse educator and promotora	individual, interpersonal	behavioral, sociocultural
**Philis-Tsimikas 2011, [44]**	San Diego, CA, USA	207/310	4	Usual care	Culturally tailored education program utilizing trained peer-educators or promotoras	individual, interpersonal	behavioral, sociocultural
**Ramal 2018, [45]**	San Bernardino County, CA, USA	38/68	6	Diabetes self-management education	Diabetes self-management education plus group support	individual, interpersonal	behavioral, sociocultural
**Rosal 2005, [46]**	Springfield, MA, USA	25/NR	6	Usual care	Culturally specific and literacy sensitive group diabetes education led by nurse, nutritionist, and assistant	individual, interpersonal	behavioral, sociocultural
**Spencer 2018, [47]**	Detroit, MI, USA	222/1049	6	Usual care	Culturally tailored diabetes self-management education, home visits, group sessions, and phone calls, delivered by CHW	individual, interpersonal	behavioral, sociocultural
**Toobert 2011, [48]**	Denver, CO, USA	280/7945	6	Usual care	Culturally adapted group lifestyle intervention targeting multiple health behaviors	individual, interpersonal	behavioral, sociocultural
**Vincent 2007, [49]**	Tucson, AZ, USA	20/60	3	Usual care	Culturally tailored self-management education and support led by promotoras	individual, interpersonal	behavioral, sociocultural
**Individual + Interpersonal/Behavioral + Health Care System**							
**McKee 2011, [50]**	Bronx, NY, USA	55/1268	6	Usual care	Health behavior counseling by home health nurses and telemetry unit for home blood pressure and glucose measurements transmitted to primary care clinicians	individual, interpersonal	behavioral, health care system
**Weinstock 2011, [51]**	New York, NY, USA; Syracuse, NY, USA	1665/NR	12	Usual care	Home telemedicine unit to videoconference with a diabetes educator for self-management education, review of home blood glucose and blood pressure measurements, and goal setting	individual, interpersonal	behavioral, health care system
**Individual + Interpersonal/Behavioral + Sociocultural + Health Care System**							
**Anderson 2010, [52]**	Middletown, CT, USA	295/1754	6	Usual care	Case management via telephone calls delivered by nurses in addition to mailed low literacy educational materials	individual, interpersonal	behavioral, sociocultural, health care system
**Babamoto 2009, [53]**	Los Angeles, CA, USA	318/1352	6	Usual care	Culturally sensitive diabetes self-management education delivered by CHWs via individual sessions and phone calls	individual, interpersonal	behavioral, sociocultural, health care system
**Pérez-Escamilla 2015, [54]**	Hartford, CT, USA	211/NR	6	Usual care	Counseling and culturally adapted diabetes education led by CHW	individual, interpersonal	behavioral, sociocultural, health care system
**Ruggiero 2014, [55]**	Chicago, IL, USA	270/888	6	Culturally sensitive educational booklet	Culturally sensitive self-care coaching by medical assistants in clinic and over the phone	individual, interpersonal	behavioral, sociocultural, health care system
**Welch 2011, [56]**	Springfield, MA, USA	46/67	12	Diabetes education materials reviewed with clinic support staff	One-on-one education sessions with diabetes nurse or dietitian using clinical decision support dashboard	individual, interpersonal	behavioral, sociocultural, health care system
**Welch 2015, [57]**	Western MA, USA	399/868	6	Usual care	One-on-one education sessions with diabetes nurse or dietitian using clinical decision support dashboard	individual, interpersonal	behavioral, sociocultural, health care system
**Individual + Interpersonal + Community/Behavioral + Sociocultural + Health Care System**							
**Baig 2015, [58]**	Chicago, IL, USA	100/211	6	One-time lecture	Culturally sensitive church-based self-management group education delivered by trained lay leader	individual, interpersonal, community	behavioral, sociocultural, health care system
**García 2015, [59]**	Central TX, USA	72/NR	6	Waitlist	Diabetes self-management education via culturally tailored home sessions by nurse	individual, interpersonal, community	behavioral, sociocultural, health care system
**Other Level/Domain Combinations**							
**Levy 2015, [60]**	New York, NY, USA	61/132	3	Usual care	Text message reminders to measure fasting blood glucose with results monitored by nurse and used for insulin titration	interpersonal	health care system
**Christian 2008, [61]**	Denver, CO, USA; Pueblo, CO, USA	310/322	12	Usual care and education materials	Computer-based assessment of diet and physical activity habits and readiness to change with tailored feedback report for patient and physician to use with motivational interviewing	individual	behavioral, health care system
**Ell 2011, [62]**	Los Angeles, CA, USA	387/1803	12	Usual care and depression educational pamphlets for patient and family	Socioculturally adapted collaborative care for depression and diabetes including psychotherapy and/or antidepressants, plus telephone symptom monitoring and relapse prevention	individual, interpersonal	sociocultural, health care system
**Seligman 2018, [63]**	Detroit, MI, USA; Houston, TX, USA; Oakland, CA, USA	568/5329	6	Waitlist	Diabetes self-management classes, individual check-ins with educator, and food delivery via food bank	individual, community	behavioral, physical

NR = not reported.

**Table 2:** Meta-analysis of effects of non-pharmacological interventions on hemoglobin A1c among U.S. Hispanic populations with type 2 diabetes, 1985–2019.

	Trials	Total N	HbA1c Weighted Mean Difference (95% Cl)	I^2^
All trials	48	9185	−0.32 (−0.44 to −0.20)	68%
>50% participants prefer Spanish	35	5888	−0.34 (−0.47 to −0.21)	51%
Single-level	23	4627	−0.25 (−0.40 to −0.10)	50%
Single-domain	12	3197	−0.32 (−0.46 to −0.18)	0%
Single-level and single-domain	9	2534	−0.34 (−0.51 to −0.17)	0%
Multi-level	25	4558	−0.39 (−0.57 to −0.21)	73%
Multi-domain	36	5988	−0.31 (−0.46 to −0.16)	74%
Multi-level and multi-domain	22	3895	−0.41 (−0.61 to −0.21)	74%
Intervention level/domain combinations *				
Individual/Behavioral	8	2492	−0.33 (−0.50 to −0.16)	0%
Individual/Behavioral + Sociocultural	13	1820	−0.24 (−0.46 to −0.01)	59%
Individual + Interpersonal/Behavioral	3	663	−0.26 (−0.55 to 0.03)	13%
Individual + Interpersonal/Behavioral + Sociocultural	10	1311	−0.54 (−0.89 to −0.19)	75%
Individual + Interpersonal/Behavioral + Health Care	2	633	−0.34 (−0.56 to −0.12)	0%
Individual + Interpersonal/Behavioral + Sociocultural + Health Care	6	1111	−0.46 (−0.85 to −0.08)	68%
Individual + Interpersonal + Community/Behavioral + Sociocultural + Health Care	2	136	−0.39 (−0.95 to 0.18)	0%

* Each of the following only had one study and therefore we did not conduct meta-analysis: Interpersonal/Health Care (Levy 2015, [60]); Individual/Behavioral + Health Care (Christian 2008, [61]); Individual + Interpersonal/Sociocultural + Health Care (Ell 2011, [62]); Individual + Community/Behavioral + Physical Environment (Seligman 2018, [63]).

## Data Availability

The original contributions presented in this study are included in the article/supplementary material. Further inquiries can be directed to the corresponding author.

## Supplementary Materials

The following supporting information can be downloaded at: https://www.mdpi.com/article/10.3390/ijerph22091345/s1, Table S1: PRISMA Checklist; Table S2: Search Terms; Table S3: Inclusion Criteria According to PICOS Framework; Table S4: Characteristics of Participants in Trials of Non-Pharmacological Interventions Among U.S. Hispanic Populations with Type 2 Diabetes, 1985–2019; Figure S1: Forest Plots; Table S5: Risk of Bias in Randomized Controlled Trials of Non-Pharmacological Interventions Among U.S. Hispanic Populations with Type 2 Diabetes, 1985–2019; Figure S2: Funnel Plots; Table S6: GRADE Strength of Evidence Assessment for Non-Pharmacological Interventions Among U.S. Hispanic Populations with Type 2 Diabetes.
