# Supplementary material for: Effects of Multilevel and Multidomain Interventions on Glycemic Control in U.S. Hispanic Populations
Source: Int J Environ Res Public Health. 2025 Aug 28;22(9):1345. doi: 10.3390/ijerph22091345 (PMC12469673; doi:10.3390/ijerph22091345)
Supplement: Supplementary file 1 [file ijerph-22-01345-s001.zip › ijerph-3806485-supplementary.pdf]

Supplemental Table S1. PRISMA Checklist

Section and Topic	Item #	Checklist item	Location where item is reported
**TITLE**			
Title	1	Identify the report as a systematic review.	Page 1
**ABSTRACT**			
Abstract	2	See the PRISMA 2020 for Abstracts checklist below.	Page 1
**INTRODUCTION**			
Rationale	3	Describe the rationale for the review in the context of existing knowledge.	Page 2
Objectives	4	Provide an explicit statement of the objective(s) or question(s) the review addresses.	Page 2
**METHODS**			
Eligibility criteria	5	Specify the inclusion and exclusion criteria for the review and how studies were grouped for the syntheses.	Page 3, Table S3
Information sources	6	Specify all databases, registers, websites, organisations, reference lists and other sources searched or consulted to identify studies. Specify the date when each source was last searched or consulted.	Page 3, Table S2
Search strategy	7	Present the full search strategies for all databases, registers and websites, including any filters and limits used.	Page 3, Table S2
Selection process	8	Specify the methods used to decide whether a study met the inclusion criteria of the review, including how many reviewers screened each record and each report retrieved, whether they worked independently, and if applicable, details of automation tools used in the process.	Page 3
Data collection process	9	Specify the methods used to collect data from reports, including how many reviewers collected data from each report, whether they worked independently, any processes for obtaining or confirming data from study investigators, and if applicable, details of automation tools used in the process.	Page 3
Data items	10a	List and define all outcomes for which data were sought. Specify whether all results that were compatible with each outcome domain in each study were sought (e.g. for all measures, time points, analyses), and if not, the methods used to decide which results to collect.	Page 3
	10b	List and define all other variables for which data were sought (e.g. participant and intervention characteristics,	Page 3

Section and Topic	Item #	Checklist item	Location where item is reported
		funding sources). Describe any assumptions made about any missing or unclear information.	
Study risk of bias assessment	11	Specify the methods used to assess risk of bias in the included studies, including details of the tool(s) used, how many reviewers assessed each study and whether they worked independently, and if applicable, details of automation tools used in the process.	Page 3
Effect measures	12	Specify for each outcome the effect measure(s) (e.g. risk ratio, mean difference) used in the synthesis or presentation of results.	Page 3
Synthesis methods	13a	Describe the processes used to decide which studies were eligible for each synthesis (e.g. tabulating the study intervention characteristics and comparing against the planned groups for each synthesis (item #5)).	Page 3
	13b	Describe any methods required to prepare the data for presentation or synthesis, such as handling of missing summary statistics, or data conversions.	Page 3
	13c	Describe any methods used to tabulate or visually display results of individual studies and syntheses.	Page 3
	13d	Describe any methods used to synthesize results and provide a rationale for the choice(s). If meta-analysis was performed, describe the model(s), method(s) to identify the presence and extent of statistical heterogeneity, and software package(s) used.	Page 3
	13e	Describe any methods used to explore possible causes of heterogeneity among study results (e.g. subgroup analysis, meta-regression).	Page 3
	13f	Describe any sensitivity analyses conducted to assess robustness of the synthesized results.	Page 3
Reporting bias assessment	14	Describe any methods used to assess risk of bias due to missing results in a synthesis (arising from reporting biases).	Page 3
Certainty assessment	15	Describe any methods used to assess certainty (or confidence) in the body of evidence for an outcome.	Page 3
**RESULTS**			
Study selection	16a	Describe the results of the search and selection process, from the number of records identified in the search to the number of studies included in the review, ideally using a flow diagram.	Page 4, Figure 1

Section and Topic	Item #	Checklist item	Location where item is reported
	16b	Cite studies that might appear to meet the inclusion criteria, but which were excluded, and explain why they were excluded.	n/a
Study characteristics	17	Cite each included study and present its characteristics.	Table 1, Table S4
Risk of bias in studies	18	Present assessments of risk of bias for each included study.	Table S5
Results of individual studies	19	For all outcomes, present, for each study: (a) summary statistics for each group (where appropriate) and (b) an effect estimate and its precision (e.g. confidence/credible interval), ideally using structured tables or plots.	Figure S1
Results of syntheses	20a	For each synthesis, briefly summarise the characteristics and risk of bias among contributing studies.	Table 1, Table S5, Table S6
	20b	Present results of all statistical syntheses conducted. If meta-analysis was done, present for each the summary estimate and its precision (e.g. confidence/credible interval) and measures of statistical heterogeneity. If comparing groups, describe the direction of the effect.	Table 2
	20c	Present results of all investigations of possible causes of heterogeneity among study results.	Page 4, Table 2
	20d	Present results of all sensitivity analyses conducted to assess the robustness of the synthesized results.	Page 4, Table 2
Reporting biases	21	Present assessments of risk of bias due to missing results (arising from reporting biases) for each synthesis assessed.	n/a
Certainty of evidence	22	Present assessments of certainty (or confidence) in the body of evidence for each outcome assessed.	Table S6
**DISCUSSION**			
Discussion	23a	Provide a general interpretation of the results in the context of other evidence.	Page 15
	23b	Discuss any limitations of the evidence included in the review.	Page 15-16
	23c	Discuss any limitations of the review processes used.	Page 15-16
	23d	Discuss implications of the results for practice, policy, and future research.	Page 16
**OTHER INFORMATION**			

Section and Topic	Item #	Checklist item	Location where item is reported
Registration and protocol	24a	Provide registration information for the review, including register name and registration number, or state that the review was not registered.	Page 3
	24b	Indicate where the review protocol can be accessed, or state that a protocol was not prepared.	Page 3
	24c	Describe and explain any amendments to information provided at registration or in the protocol.	n/a
Support	25	Describe sources of financial or non-financial support for the review, and the role of the funders or sponsors in the review.	Page 16
Competing interests	26	Declare any competing interests of review authors.	Page 16
Availability of data, code and other materials	27	Report which of the following are publicly available and where they can be found: template data collection forms; data extracted from included studies; data used for all analyses; analytic code; any other materials used in the review.	Page 16

PRISMA Checklist for Abstracts

Section and Topic	Item #	Checklist item	Reported (Yes/No)
**TITLE**			
Title	1	Identify the report as a systematic review.	Yes
**BACKGROUND**			
Objectives	2	Provide an explicit statement of the main objective(s) or question(s) the review addresses.	Yes
**METHODS**			
Eligibility criteria	3	Specify the inclusion and exclusion criteria for the review.	Yes
Information sources	4	Specify the information sources (e.g. databases, registers) used to identify studies and the date when each was last searched.	Yes
Risk of bias	5	Specify the methods used to assess risk of bias in the included studies.	Yes
Synthesis of results	6	Specify the methods used to present and synthesise results.	Yes
**RESULTS**			

Section and Topic	Item #	Checklist item	Reported (Yes/No)
Included studies	7	Give the total number of included studies and participants and summarise relevant characteristics of studies.	Yes
Synthesis of results	8	Present results for main outcomes, preferably indicating the number of included studies and participants for each. If meta-analysis was done, report the summary estimate and confidence/credible interval. If comparing groups, indicate the direction of the effect (i.e. which group is favoured).	Yes
**DISCUSSION**			
Limitations of evidence	9	Provide a brief summary of the limitations of the evidence included in the review (e.g. study risk of bias, inconsistency and imprecision).	Yes
Interpretation	10	Provide a general interpretation of the results and important implications.	Yes
**OTHER**			
Funding	11	Specify the primary source of funding for the review.	Yes
Registration	12	Provide the register name and registration number.	Yes

From: Page MJ, McKenzie JE, Bossuyt PM, Boutron I, Hoffmann TC, Mulrow CD, et al. The PRISMA 2020 statement: an updated guideline for reporting systematic reviews. BMJ 2021;372:n71. doi: 10.1136/bmj.n71

Supplemental Table S2. Search Terms

DATABASE	CONCEPT	SEARCH TERMS
**CINAHL**<br>*3/1/2019: 27,473 RESULTS*	*Diabetes*	((((((MH "Diabetes Mellitus+") OR (MH "Diabetes Education") OR (MH "Diabetes Educators") OR diabetes OR diabetic OR diabetics OR "noninsulin-dependent" OR "noninsulin dependent" OR "non-insulin-dependent" OR "non-insulin dependent") NOT ((MH "Diabetes Insipidus") OR "diabetes insipidus"))
	Study Design	AND (LA English) AND (PY 1996-2019) NOT ((TI "meta-analysis" OR TI "cross-sectional"))) NOT (PT systematic review OR PT doctoral dissertation OR PT masters thesis OR PT proceedings OR PT anecdote)
	Language	AND (language OR languages OR English OR literacy OR "foreign-born" OR "foreign born" OR immigrant OR immigrants OR refugee OR refugees OR migrant OR migrants OR interpreter OR interpreters OR monolingual OR bilingual OR multilingual OR linguistic OR linguistically OR (MH "English as a Second Language") OR (MH "Language") OR (MH "Literacy") OR (MH "Illiteracy")
	Race/Ethnicity	OR (MH "Immigrants+") OR (MH "Refugees") OR (MH "Race Relations+") OR (MH "Race Factors") OR (MH "Minority Groups") OR (MH "Ethnic Groups") OR (MH "Arabs") OR (MH "Asians+") OR (MH "Blacks") OR (MH "Hispanics") OR (MH "Eskimos+") OR (MH "Native Americans") OR (MH "Health Services, Indigenous") OR (MH "Transcultural Care") OR (MH "Cultural Competence") OR (MH "Cultural Sensitivity") OR cultural OR culturally OR multicultural OR multi-cultural OR transcultural OR trans-cultural OR crosscultural OR cross-cultural OR ethnic OR ethnicity OR ethnicities OR ethnically OR multiethnic OR multi-ethnic OR race OR races OR racial OR racially OR multiracial OR multi-racial OR biracial OR minority OR minorities OR "non-white" OR "non-whites" OR Black OR Blacks OR African OR Hispanic OR Hispanics OR Latino OR Latinos OR Latina OR Latinas OR Latinx OR "Puerto Rican" OR "Puerto Ricans" OR "Puerto Rico" OR "Mexican American" OR "Mexican Americans" OR "Cuban American" OR "Cuban Americans" OR "Native American" OR "Native Americans" OR "American Indian" OR "American Indians" OR "Indian Health Service" OR "Alaska native" OR "Alaska natives" OR "Alaskan native" OR "Alaskan natives" OR "native Hawaiian" OR "native Hawaiians" OR "pacific islander" OR "pacific islanders" OR "American Samoan" OR "American Samoans" OR "American Samoa" OR Guam OR Guamanian OR Guamanians OR Chamorro OR Chamorros OR "Arab American" OR "Arab Americans" OR Asian OR Asians OR "Chinese American" OR "Chinese Americans" OR "Filipino American" OR "Filipino Americans" OR "Indian American" OR "Indian Americans" OR "people of color"
	Disparities	OR (MH "Healthcare Disparities") OR (MH "Health Status Disparities") OR (MH "Health Services Accessibility") OR (MH "Social Determinants of Health") OR

DATABASE	CONCEPT	SEARCH TERMS
		(MH "Community Health Centers") OR (MH "Safety-Net Providers") OR (MH "Community Health Services") OR (MH "Medically Underserved") OR (MH "Medically Underserved Area") OR (MH "Poverty+") OR (MH "Health Services for the Indigent") OR (MH "Urban Health Services") OR (MH "Medically Uninsured") OR equity OR inequity OR inequities OR equality OR inequality OR inequalities OR disparity OR disparities OR poor OR poverty OR vulnerable OR indigent OR underserved OR disadvantaged OR uninsured OR "publicly insured" OR Medicaid OR "safety-net" OR "safety net" OR "health center" OR FQHC OR "community clinic" OR "free clinic" OR "low income" OR socioeconomic OR "social determinants")))
**PSYCINFO** ***3/1/2019: 7,150 RESULTS***	*Diabetes*	(((SU "Diabetes Mellitus") OR (SU "Type 2 Diabetes") OR diabetes OR diabetic OR diabetics OR "noninsulin-dependent" OR "noninsulin dependent" OR "non-insulin-dependent" OR "non-insulin dependent") AND PY 1996-2019 AND LA English NOT ((SU "Diabetes Insipidus") OR "diabetes insipidus")
	Study Design	NOT (TI "meta-analysis" OR TI "cross-sectional" OR MR "meta analysis" OR MR "meta synthesis" OR MR "systematic review" OR MR "scientific simulation" OR PT "dissertation abstract" OR (PT "encyclopedia") OR (PZ "abstract collection") OR (PZ "bibliography") OR (PZ "dissertation") OR (PZ "encyclopedia entry") OR (PZ "interview") OR (PZ "obituary") OR (PZ "poetry") OR (PZ "review-book") OR (PZ "review-media") OR (PZ "review-software & other") OR (PZ "column/opinion") or (PZ "editorial") OR (PZ "comment/reply") or (PZ "erratum/correction") OR (PZ "publication information")) NOT ((PO Animal NOT (PO Animal AND PO Human))))
	Language	AND (language OR languages OR (SU "English as Second Language") OR (SU "Language Proficiency") OR "English proficiency" OR "English speaking" OR "non-English" OR (SU "Literacy") OR literacy OR monolingual OR bilingual OR multilingual OR linguistic OR linguistically OR (SU "Interpreters") OR interpreter OR interpreters OR foreign-born OR "foreign born"
	Race/Ethnicity	OR immigrant OR immigrants OR refugee OR refugees OR (SU "Migrant Farm Workers") OR (SU "Blacks") OR (SU "African Cultural Groups") OR black OR blacks OR African OR (SU "Alaska Natives") OR (SU "American Indians") OR "native American" OR "native Americans" OR "American Indian" OR "American Indians" OR "Indian Health Service" OR "Alaska native" OR "Alaska natives" OR "Alaskan native" OR "Alaskan natives" OR (SU "Arabs") OR "Arab American" OR "Arab Americans" OR (SU "Asians") OR Asian OR Asians OR "Chinese American" OR "Chinese Americans" OR "Filipino American" OR "Filipino Americans" OR "Indian American" OR "Indian Americans" OR (SU "Chinese Cultural Groups") OR (SU "Southeast Asian Cultural Groups") OR (SU "South Asian Cultural Groups") OR (SU "Latinos/Latinas") OR (SU "Mexican Americans") OR Hispanic OR Hispanics OR Latino OR Latinos OR

DATABASE	CONCEPT	SEARCH TERMS
		Latina OR Latinas OR Latinx OR "Puerto Rican" OR "Puerto Ricans" OR "Puerto Rico" OR "Mexican American" OR "Mexican Americans" OR "Cuban American" OR "Cuban Americans" OR (SU "Pacific Islanders") OR (SU "Hawaii Natives") OR "native Hawaiian" OR "native Hawaiians" OR "pacific islander" OR "pacific islanders" OR "American Samoan" OR "American Samoans" OR "American Samoa" OR Guam OR Guamanian OR Guamanians OR Chamorro OR Chamorros OR (SU "Racial and Ethnic Groups") OR ethnic OR ethnicity OR ethnicities OR ethnically OR multiethnic OR multi-ethnic OR race OR races OR racial OR racially OR multiracial OR multi-racial OR biracial OR minority OR minorities OR "non-white" OR "non-whites" OR "people of color"
	Disparities	OR (SU "Cultural Sensitivity") OR (SU "Cross Cultural Treatment") OR (SU "Cross Cultural Communication") OR cultural OR culturally OR multicultural OR multi-cultural OR (SU "Health Disparities") OR equity OR inequity OR inequities OR equality OR inequality OR inequalities OR disparity OR disparities OR "health center" OR FQHC OR (SU "Community Health") OR "community clinic" OR "free clinic" OR "safety-net" OR "safety net" OR uninsured OR "publicly insured" OR Medicaid OR poor OR poverty OR indigent OR "low income" OR socioeconomic OR "urban health" OR vulnerable OR underserved OR disadvantaged OR "social determinants")
**PUBMED** ***3/1/2019: 36,969 RESULTS***	*Diabetes*	"Diabetes Mellitus"[Mesh] OR diabetes[tiab] OR diabetic[tiab] OR diabetics[tiab] OR "noninsulin-dependent"[tiab] OR "noninsulin dependent"[tiab] OR "non-insulin-dependent"[tiab] OR "non-insulin dependent"[tiab]) NOT ("Diabetes Insipidus"[Mesh] or "diabetes insipidus"
	Study Design	NOT ((("Animals"[Mesh] NOT ("Animals"[Mesh] AND "Humans"[Mesh]))))) AND "1996/01/01"[PDat] : "3000/12/31"[PDat])) NOT (meta-analysis[ti] or "cross-sectional"[ti])) NOT ((Address[pt] or Autobiography[pt] or Bibliography[pt] or Biography[pt] or "Case Reports"[pt] or "Clinical Conference"[pt] or "Clinical Trial, Veterinary"[pt] or "Clinical Trial Protocol"[pt] or Congress[pt] or "Consensus Development Conference"[pt] or dataset[pt] or "Consensus Development Conference, NIH"[pt] or "Dictionary"[pt] or "Dictionary"[pt] or "Duplicate Publication"[pt] or Editorial[pt] or "Expression of Concern"[pt] or Festschrift[pt] or "Government Document"[pt] or Guideline[pt] or "Interactive Tutorial"[pt] or Interview[pt] or Lecture[pt] or "Legal Case"[pt] or Legislation[pt] or "Meta-Analysis"[pt] or news[pt] or "Newspaper Article"[pt] or "Observational Study, Veterinary"[pt] or "Patient Education Handout"[pt] or Personal Narrative[pt] or Portrait[pt] or Practice Guideline[pt] or "Publication Components"[pt] or review[pt] or "Scientific Integrity Review"[pt] or "Study Characteristics"[pt] or "Systematic Review"[pt] or "Video-Audio Media"[pt] or Webcasts[pt])))) AND English[lang]

DATABASE	CONCEPT	SEARCH TERMS
	Race/Ethnicity	OR "Refugees"[Mesh] OR ("Ethnic Groups"[Mesh:NoExp] OR "African Americans"[Mesh] OR "American Native Continental Ancestry Group"[Mesh] OR "Asian Americans"[Mesh] OR "Oceanic Ancestry Group"[Mesh] OR "Arabs"[Mesh] OR "Hispanic Americans"[Mesh] OR "Race Factors"[Mesh] OR "Cultural Competency"[Mesh] OR "Culturally Competent Care"[Mesh] OR cultural[tw] OR culturally[tw] OR multicultural[tw] OR multi-cultural[tw] OR transcultural[tw] OR trans-cultural[tw] OR crosscultural[tw] OR cross-cultural[tw] OR ethnic[tw] OR ethnicity[tw] OR ethnicities[tw] OR ethnically [tw] OR multiethnic[tw] OR multi-ethnic[tw] OR race[tw] OR races[tw] OR racial[tw] OR racially[tw] OR multiracial[tw] OR multi-racial[tw] OR biracial[tw] OR minority[tw] OR minorities[tw] OR "non-white"[tw] OR "non-whites"[tw] OR black[tw] OR blacks[tw] OR African[tw] OR Hispanic[tw] OR Hispanics[tw] OR Latino[tw] OR Latinos[tw] OR Latina[tw] OR Latinas[tw] OR Latinx[tw] OR "Puerto Rican"[tw] OR "Puerto Ricans"[tw] OR "Puerto Rico"[tw] OR "Mexican American"[tw] OR "Mexican Americans"[tw] OR "Cuban American"[tw] OR "Cuban Americans"[tw] OR "native American"[tw] OR "native Americans"[tw] OR "American Indian"[tw] OR "American Indians" OR "Indian health service"[tw] OR "United States Indian Health Service"[Mesh] OR "Alaska native"[tw] OR "Alaska natives"[tw] OR "Alaskan native"[tw] OR "Alaskan natives"[tw] OR "native Hawaiian"[tw] OR "native Hawaiians"[tw] OR "pacific islander"[tw] OR "pacific islanders"[tw] OR "American Samoan"[tw] OR "American Samoa"[tw] OR "American Samoans"[tw] OR Guam[tw] OR Guamanian[tw] OR Guamanians[tw] OR Chamorro[tw] OR Chamorros[tw] OR "Arab American"[tw] OR "Arab Americans"[tw] OR Asian[tw] OR Asians[tw] OR "Chinese American"[tw] OR "Filipino American"[tw] OR "Indian American"[tw] OR "people of color"[tw]))
	Language	AND ((((Language[Mesh] OR language[tw] OR languages[tw] OR English[tw] OR Literacy[Mesh] OR literacy[tw] OR "foreign-born"[tw] OR "foreign born"[tw] OR immigrant[tw] OR immigrants[tw] OR refugee[tw] OR refugees[tw] OR interpreter[tw] OR interpreters[tw] OR monolingual[tw] OR bilingual[tw] OR multilingual[tw] OR linguistic[tw] OR linguistically[tw]))
	Disparities	OR (Healthcare Disparities[Mesh] OR "Health Status Disparities"[Mesh] OR "Health Equity"[Mesh] OR "Social Determinants of Health"[Mesh] OR "Medically Underserved Area"[Mesh] OR "Medically Uninsured"[Mesh] OR "Health Services Accessibility"[Mesh:NoExp] OR "Urban Health"[Mesh] OR "Community Health Services"[Mesh:NoExp] OR "Community Health Centers"[Mesh:NoExp] OR "Safety-net Providers"[Mesh] OR "Poverty"[Mesh] OR equity[tw] OR inequity[tw] OR inequities[tw] OR equality[tw] OR inequality[tw] OR inequalities[tw] OR disparity[tw] OR disparities[tw] OR poor[tw] OR poverty[tw] OR vulnerable[tw] OR indigent[tw] OR underserved[tw] OR disadvantaged[tw] OR uninsured[tw] OR "publicly insured"[tw] OR

DATABASE	CONCEPT	SEARCH TERMS
		Medicaid[tw] OR "safety-net"[tw] OR "safety net"[tw] OR "health center"[tw] OR FQHC[tw] OR "community clinic"[tw] OR "free clinic"[tw] OR "low income"[tw]))))
**SCOPUS** ***3/1/2019: 39,697 RESULTS***	*Diabetes*	((TITLE-ABS-KEY ("diabetes") OR TITLE-ABS-KEY ("diabetic") OR TITLE-ABS-KEY ("noninsulin-dependent") OR TITLE-ABS-KEY ("T2D") OR TITLE-ABS-KEY ("non-insulin-dependent")) AND NOT (TITLE-ABS-KEY ("diabetes insipidus"))
	Study Design	AND NOT (TITLE ("meta-analysis") OR TITLE ("cross-sectional")) AND NOT (DOCTYPE (ab) OR DOCTYPE (bk) OR DOCTYPE (ch) OR DOCTYPE (cr) OR DOCTYPE (er)) AND NOT ((KEY (animal OR nonhuman)) AND NOT (KEY (animal OR nonhuman) AND KEY (human)))) AND (LIMIT-TO (PUBYEAR , 2019) OR LIMIT-TO (PUBYEAR , 2018) OR LIMIT-TO (PUBYEAR , 2017) OR LIMIT-TO (PUBYEAR , 2016) OR LIMIT-TO (PUBYEAR , 2015) OR LIMIT-TO (PUBYEAR , 2014) OR LIMIT-TO (PUBYEAR , 2013) OR LIMIT-TO (PUBYEAR , 2012) OR LIMIT-TO (PUBYEAR , 2011) OR LIMIT-TO (PUBYEAR , 2010) OR LIMIT-TO (PUBYEAR , 2009) OR LIMIT-TO (PUBYEAR , 2008) OR LIMIT-TO (PUBYEAR , 2007) OR LIMIT-TO (PUBYEAR , 2006) OR LIMIT-TO (PUBYEAR , 2005) OR LIMIT-TO (PUBYEAR , 2004) OR LIMIT-TO (PUBYEAR , 2003) OR LIMIT-TO (PUBYEAR , 2002) OR LIMIT-TO (PUBYEAR , 2001) OR LIMIT-TO (PUBYEAR , 2000) OR LIMIT-TO (PUBYEAR , 1999) OR LIMIT-TO (PUBYEAR , 1998) OR LIMIT-TO (PUBYEAR , 1997) OR LIMIT-TO (PUBYEAR , 1996))
	Language	AND (TITLE-ABS-KEY ("language") OR TITLE-ABS-KEY ("English") OR TITLE-ABS-KEY ("literacy") OR TITLE-ABS-KEY ("monolingual") OR TITLE-ABS-KEY ("bilingual") OR TITLE-ABS-KEY ("multilingual") OR TITLE-ABS-KEY ("linguistic") OR TITLE-ABS-KEY ("linguistically") OR TITLE-ABS-KEY ("interpreter") AND (LIMIT-TO (LANGUAGE , "English") OR EXCLUDE (LANGUAGE , "Spanish") OR EXCLUDE (LANGUAGE , "Portuguese") OR EXCLUDE (LANGUAGE , "French") OR EXCLUDE (LANGUAGE , "Turkish") OR EXCLUDE (LANGUAGE , "German") OR EXCLUDE (LANGUAGE , "Italian") OR EXCLUDE (LANGUAGE , "Polish") OR EXCLUDE (LANGUAGE , "Arabic") OR EXCLUDE (LANGUAGE , "Chinese") OR EXCLUDE (LANGUAGE , "Croatian") OR EXCLUDE (LANGUAGE , "Thai") OR EXCLUDE (LANGUAGE , "Dutch") OR EXCLUDE (LANGUAGE , "Korean") OR EXCLUDE (LANGUAGE , "Japanese") OR EXCLUDE (LANGUAGE , "Russian") OR EXCLUDE (LANGUAGE , "Serbian") OR EXCLUDE (LANGUAGE , "Czech") OR EXCLUDE (LANGUAGE , "Persian") OR EXCLUDE (LANGUAGE ,

DATABASE	CONCEPT	SEARCH TERMS
		"Slovak") OR EXCLUDE (LANGUAGE , "Greek") OR EXCLUDE (LANGUAGE , "Romanian") OR EXCLUDE (LANGUAGE , "Slovenian")) AND (LIMIT-TO (AFFILCOUNTRY , "United States") OR LIMIT-TO (AFFILCOUNTRY , "Puerto Rico") OR LIMIT-TO (AFFILCOUNTRY , "American Samoa") OR LIMIT-TO (AFFILCOUNTRY , "Guam") OR LIMIT-TO (AFFILCOUNTRY , "Virgin Islands (U.S.)") OR LIMIT-TO (AFFILCOUNTRY , "Undefined"))
	Race/Ethnicity	OR TITLE-ABS-KEY ("foreign-born") OR TITLE-ABS-KEY ("immigrant") OR TITLE-ABS-KEY ("refugee") OR TITLE-ABS-KEY ("migrant") OR TITLE-ABS-KEY ("black") OR TITLE-ABS-KEY ("African") OR TITLE-ABS-KEY ("native American") OR TITLE-ABS-KEY ("American Indian") OR TITLE-ABS-KEY ("Alaska native") OR TITLE-ABS-KEY ("Alaskan native") OR TITLE-ABS-KEY ("Indian health service") OR TITLE-ABS-KEY ("Arab American") OR TITLE-ABS-KEY ("Asian") OR TITLE-ABS-KEY ("Chinese American") OR TITLE-ABS-KEY ("Filipino American") OR TITLE-ABS-KEY ("Indian American") OR TITLE-ABS-KEY ("Hispanic") OR TITLE-ABS-KEY ("Latino") OR TITLE-ABS-KEY ("Latina") OR TITLE-ABS-KEY ("Latinx") OR TITLE-ABS-KEY ("Puerto Rican") OR TITLE-ABS-KEY ("Puerto Rico") OR TITLE-ABS-KEY ("Mexican American") OR TITLE-ABS-KEY ("Cuban American") OR TITLE-ABS-KEY ("native Hawaiian") OR TITLE-ABS-KEY ("pacific islander") OR TITLE-ABS-KEY ("American Samoa") OR TITLE-ABS-KEY ("American Samoan") OR TITLE-ABS-KEY ("Guam") OR TITLE-ABS-KEY ("Guamanian") OR TITLE-ABS-KEY ("Chamorro") OR TITLE-ABS-KEY ("ethnic") OR TITLE-ABS-KEY ("ethnicity") OR TITLE-ABS-KEY ("ethnicities") OR TITLE-ABS-KEY ("ethnically ") OR TITLE-ABS-KEY ("multiethnic") OR TITLE-ABS-KEY ("race") OR TITLE-ABS-KEY ("racial") OR TITLE-ABS-KEY ("racially") OR TITLE-ABS-KEY ("multiracial") OR TITLE-ABS-KEY ("biracial") OR TITLE-ABS-KEY ("minority") OR TITLE-ABS-KEY ("minorities") OR TITLE-ABS-KEY ("non-white") OR TITLE-ABS-KEY ("people of color")
	Disparities	OR TITLE-ABS-KEY ("cultural") OR TITLE-ABS-KEY ("culturally") OR TITLE-ABS-KEY ("multicultural") OR TITLE-ABS-KEY ("transcultural") OR TITLE-ABS-KEY ("crosscultural") OR TITLE-ABS-KEY ("equity") OR TITLE-ABS-KEY ("inequity") OR TITLE-ABS-KEY ("inequities") OR TITLE-ABS-KEY ("equality") OR TITLE-ABS-KEY ("inequality") OR TITLE-ABS-KEY ("inequalities") OR TITLE-ABS-KEY ("disparity") OR TITLE-ABS-KEY ("disparities") OR TITLE-ABS-KEY ("safety-net") OR TITLE-ABS-KEY ("health center") OR TITLE-ABS-KEY ("FQHC") OR TITLE-ABS-KEY ("community clinic") OR TITLE-ABS-KEY ("free clinic") OR TITLE-ABS-KEY ("uninsured") OR TITLE-ABS-KEY ("publicly insured") OR TITLE-ABS-

DATABASE	CONCEPT	SEARCH TERMS
		KEY ("Medicaid") OR TITLE-ABS-KEY ("poor") OR TITLE-ABS-KEY ("poverty") OR TITLE-ABS-KEY ("indigent") OR TITLE-ABS-KEY ("low income") OR TITLE-ABS-KEY ("urban health") OR TITLE-ABS-KEY ("vulnerable") OR TITLE-ABS-KEY ("underserved") OR TITLE-ABS-KEY ("disadvantaged") OR TITLE-ABS-KEY ("social determinants"))

Supplemental Table S3. Inclusion Criteria According to PICOS Framework

	Criteria
Population	Adults ≥ 18 years<br>Type 2 diabetes<br>USA including territories<br>≥ 50% Hispanic/Latino (or stratified results for Hispanic/Latino participants)
Intervention	Any non-pharmacological intervention*
Comparison	Any control comparison group (e.g., usual care, attention control, waitlist)
Outcome	Hemoglobin A1C
Study Design	Randomized controlled trial<br>≥ 3 months duration

*Based on NIH definition: An intervention is defined as a manipulation of the subject or subject's environment for the purpose of modifying one or more health-related biomedical or behavioral processes and/or endpoints. Examples include: delivery systems (e.g., telemedicine, face-to-face interviews); strategies to change health-related behavior (e.g., cognitive therapy, exercise, development of new habits); and treatment strategies. From https://grants.nih.gov/faqs#/clinical-trial-definition.htm?anchor=54901. We excluded inpatient, nursing home, and emergency department based interventions; drugs, devices, surgeries, procedures, and diets; and interventions focused on diabetes prevention, screening, or diagnosis.

Supplemental Table S4. Characteristics of Participants in Trials of Non-Pharmacological Interventions Among U.S. Hispanic Populations with Type 2 Diabetes, 1985-2019

Study	Arm	N	Female	Hispanic	Specific Populations	Age
Anderson 2010, [52]	Control	149	57%	78%	Puerto Rican	NR
	Intervention	146	59%	73%	Puerto Rican	NR
Aponte 2017, [16]	Control	60	65%	100%	20% Puerto Rican<br>20% Mexican<br>40% Dominican<br>10% Honduran<br>5% Ecuadorian<br>5% Guatemalan or El Salvadorian	Mean 59 (SD 8)
	Intervention	60	80%	100%	30% Puerto Rican<br>20% Mexican<br>20% Dominican<br>15% Honduran<br>5% Guatemalan or El Salvadorian	Mean 59 (SD 9)
Ayala 2015, [37]	Control	155	61%	95%	Targeted Mexican Americans	Mean 56 (SD 12)
	Intervention	149	66%	97%	Targeted Mexican Americans	Mean 57 (SD 12)
Babamoto 2009, [53]	Control	54	78%	100%	NR	Mean 50 (SD 11)
	Intervention	75	64%	100%	NR	Mean 51 (SD 13)
Baig 2015, [58]	Control	50	80%	100%	92% Mexican origin	Mean 56 (SD 11)
	Intervention	50	82%	96%	98% Mexican origin	Mean 52 (SD 12)
Brown 2002, [40]	Control	126	68%	100%	Mexican American	Mean 53 (SD 8)
	Intervention	126	60%	100%	Mexican American	Mean 55 (SD 8)
Brown 2005, [41]	Control	114	61%	100%	Mexican American	Mean 50 (SD 8)
	Intervention	102	60%	100%	Mexican American	Mean 50 (SD 8)
Brown 2011, [42]	Control	35	74%	100%	Mexican American	Mean 50 (SD 9)
	Intervention	48	65%	100%	Mexican American	Mean 49 (SD 8)
Burner 2018, [38]	Control	22	64%	82%	NR	Mean 46 (SD 9)
	Intervention	22	50%	77%	NR	Mean 46 (SD 10)
Carrasquillo 2017, [24]	Control	150	55%	100%	NR	Mean 55 (SD 6)

Study	Arm	N	Female	Hispanic	Specific Populations	Age
	Intervention	150	55%	100%	NR	Mean 55 (SD 7)
Castaneda 2002, [17]	Control	31	61%	100%	84% Caribbean<br>10% Central American and Panamanian<br>6% South American	Mean 66 (SE 1)
	Intervention	31	68%	100%	90% Caribbean<br>7% Central American and Panamanian<br>3% South American	Mean 66 (SE 2)
Castejón 2013, [25]	Control	24	79%	100%	33% Caribbean<br>13% Central American<br>42% South American<br>13% Other	Mean 55 (SD 10)
	Intervention	19	58%	100%	11% Caribbean<br>11% Central American<br>74% South American<br>5% Other	Mean 54 (SD 9)
Chamany 2015, [18]	Control	498	63%	69%	NR	Mean 56 (SD 12)
	Intervention	443	65%	66%	NR	Mean 57 (SD 11)
Christian 2008, [61]	Control	155	68%	100%	NR	Mean 53 (SD 11)
	Intervention	155	65%	100%	NR	Mean 53 (SD 11)
Ell 2011, [62]	Control	194	82%	96%	Country of origin:<br>83% Mexico<br>13% Central America	Mean 54 (SD 9)
	Intervention	193	82%	96%		Mean 54 (SD 9)
Fortmann 2017; [26]	Control	63	76%	100%	Country of origin:<br>89% Mexico<br>6% US<br>5% Other	Mean 49 (SD 11)
	Intervention	63	73%	100%	Country of origin:<br>93% Mexico<br>3% US<br>3% Other	Mean 48 (SD 9)
Frosch 2011; [19]	Control	101	43%	55%	NR	Mean 54 (SD 9)
	Intervention	100	54%	57%	NR	Mean 57 (SD 8)
García 2014, [59]	Control	33	73%	100%	Targeted Mexican Americans	Mean 49 (SD 10)
	Intervention	39	62%	100%	Targeted Mexican Americans	Mean 50 (SD 9)
Gerber 2005, [27]	Control (Higher Literacy)	55	66%	55%	NR	Mean 52 (SD 11)

Study	Arm	N	Female	Hispanic	Specific Populations	Age
	Control (Lower Literacy)	67	60%	72%	NR	Mean 60 (SD 11)
	Intervention (Higher Literacy)	54	76%	56%	NR	Mean 49 (SD 12)
	Intervention (Lower Literacy)	68	65%	78%	NR	Mean 58 (SD 12)
Heisler 2014, [28]	Control	95	66%	61%	NR	Mean 52 (SD 9)
	Intervention	93	76%	53%	NR	Mean 51 (SD 9)
Khanna 2014, [29]	Control	37	32%	100%	NR	Mean 53 (SD 12)
	Intervention	38	50%	100%	NR	Mean 51 (SD 12)
Levy 2015, [60]	Control	28	57%	61%	NR	Mean 45 (SD 10)
	Intervention	33	45%	55%	NR	Mean 48 (SD 11)
Lorig 2008, [39]	Control	198	67%	100%	76% Born in Mexico	Mean 53 (SD 13)
	Intervention	219	57%	100%	69% Born in Mexico	Mean 53 (SD 13)
Lujan 2007, [30]	Intervention & Control	150	80%	100%	100% Mexican American	Mean 58
McEwen 2017, [43]	Control	74	72%	100%	100% Mexican American	Mean 53 (SD 8)
	Intervention	83	59%	100%	100% Mexican American	Mean 54 (SD 10)
McKee 2011, [50]	Control	24	67%	75%	NR	Mean 59 (SD 8)
	Intervention	31	67%	71%	NR	Mean 61 (SD 11)
Moncrieft 2016, [20]	Control	54	78%	78%	NR	Mean 55 (SD 6)
	Intervention	57	65%	91%	NR	Mean 55 (SD 8)
Noël 1998, [21]	Intervention & Control	596	63%	85%	Mostly Mexican American	Mean 51 (SD 11)
Osborn 2010, [31]	Control	43	70%	100%	100% Puerto Rican	Mean 58 (SD 10)
	Intervention	48	79%	100%	100% Puerto Rican	Mean 57 (SD 11)
Palmas 2014, [22]	Control	179	63%	100%	Mainly Dominican origin	Mean 58 (SD 8)
	Intervention	181	61%	100%	Mainly Dominican origin	Mean 57 (SD 8)

Study	Arm	N	Female	Hispanic	Specific Populations	Age
Pérez-Escamilla 2015, [54]	Control	106	75%	100%	Puerto Rican/Dominican targeted	Mean 57 (SD 12)
	Intervention	105	72%	100%	Puerto Rican/Dominican targeted	Mean 55 (SD 12)
Philis-Tsimikas 2011, [44]	Control	103	75%	100%	100% Mexican American	Mean 49 (SD 12)
	Intervention	104	66%	100%	100% Mexican American	Mean 52 (SD 10)
Prezio 2013, [32]	Control	90	54%	82%	Majority Mexican American	Mean 46 (SD 11)
	Intervention	90	67%	81%	Majority Mexican American	Mean 48 (SD 11)
Ramal 2018, [45]	Control	19	80%	100%	Country of origin:<br>80% Mexico<br>13% Other North American countries<br>7% South America	Mean 53 (SD 13)
	Intervention	19	77%	100%	Country of origin:<br>77% Mexico<br>12% South America	Mean 53 (SD 7)
Rosal 2005, [46]	Control	10	80%	100%	Puerto Rican	Mean 62 (SD 10)
	Intervention	15	80%	100%	Puerto Rican	Mean 63 (SD 8)
Rosal 2011, [33]	Control	128	75%	100%	86% Birthplace Puerto Rico	18-44 years: 17%<br>45-54 years: 27%<br>55-64 years: 37%<br>65+ years: 19%
	Intervention	124	78%	100%	90% Birthplace Puerto Rico	18-44 years: 15%<br>45-54 years: 32%<br>55-64 years: 29%<br>65+ years: 23%
Rothschild 2014, [34]	Control	71	70%	100%	100% Mexican American	Mean 54 (SD 13)
	Intervention	73	64%	100%	100% Mexican American	Mean 54 (SD 12)
Ruggiero 2014, [55]	Control	132	70%	46%*	NR	Mean 53 (SD 13)
	Intervention	134	68%	47%*	NR	Mean 53 (SD 12)
Seligman 2018, [63]	Control	283	67%	52%	NR	Mean 55 (SD 12)

Study	Arm	N	Female	Hispanic	Specific Populations	Age
	Intervention	285	70%	53%	NR	Mean 55 (SD 11)
Sixta 2008, [35]	Control	68	71%	100%	100% Mexican American	Mean 53 (range 26-81)
	Intervention	63	71%	100%	100% Mexican American	Mean 55 (range 30-77)
Spencer 2018, [47]	Control	73	67%	100%	NR	Mean 49 (SD 10)
	Intervention	89	61%	100%	NR	Mean 48 (SD 11)
	Intervention	60	53%	100%	NR	Mean 50 (SD 11)
Sugiyama 2015, [23]	Control	258	73%	59%	NR	Mean 63 (SD 7)
	Intervention	258	69%	64%	NR	Mean 64 (SD 6)
Toobert 2011, [48]	Control	138	100%	100%	NR	Mean 59 (SD 10)
	Intervention	142	100%	100%	NR	Mean 56 (SD 10)
Vincent 2007, [49]	Control	8	50%	100%	100% Mexican American	Mean 55 (SD 8)
	Intervention	9	89%	100%	100% Mexican American	Mean 57 (SD 11)
Wagner 2016, [36]	Control	46	72%	100%	NR	Mean 61 (SD 12)
	Intervention	61	74%	100%	NR	Mean 60 (SD 11)
Weinstock 2011, [51]	Intervention & Control	585	68%	100%	NR	Mean 70 (SD 6)
Welch 2011, [56]	Control	21	62%	100%	Predominantly Puerto Rican	Mean 58 (SD 10)
	Intervention	25	68%	100%	Predominantly Puerto Rican	Mean 54 (SD 10)
Welch 2015, [57]	Control	200	59%	100%	NR	Mean 55 (SD 12)
	Intervention	199	61%	100%	NR	Mean 55 (SD 10)

NR=not reported
* % Hispanic in overall study population; subgroup results reported for Hispanic participants were included in meta-analyses

Supplemental Figure S1. Forest Plots

a. All trials

Author	Mean Difference (95% CI)
Anderson 2010	-0.01 [-0.33; 0.31]
Aponte 2017	-0.60 [-1.47; 0.27]
Ayala 2015	0.00 [-0.46; 0.46]
Babamoto 2009	-0.20 [-0.98; 0.58]
Baig 2015	-0.21 [-0.97; 0.55]
Brown 2002	-1.40 [-2.15; -0.65]
Brown 2005	0.10 [-0.77; 0.97]
Brown 2011	1.80 [0.61; 2.99]
Burner 2018	-0.80 [-2.07; 0.47]
Carrasquillo 2017	-0.59 [-1.08; -0.10]
Castaneda 2004	-0.70 [-1.76; 0.36]
Castejon 2013	-0.70 [-1.38; -0.02]
Chamany 2015	-0.40 [-0.74; -0.06]
Christian 2008	0.32 [-0.09; 0.73]
Ell 2011	0.01 [-0.49; 0.51]
Fortmann 2017	-0.90 [-1.53; -0.27]
Frosch 2011	-0.30 [-0.83; 0.23]
Garcia 2014	-0.60 [-1.44; 0.24]
Gerber 2005a	-0.10 [-0.77; 0.57]
Gerber 2005b	0.80 [0.12; 1.48]
Heisler 2014	-0.10 [-0.40; 0.20]
Khanna 2014	0.20 [-0.37; 0.77]
Levy 2015	-0.85 [-1.83; 0.13]
Lorig 2008	-0.36 [-0.67; -0.05]
Lujan 2008	-0.25 [-0.86; 0.36]
McEwen 2017	-0.55 [-1.26; 0.16]
McKee 2011	-0.60 [-1.86; 0.66]
Moncrieft 2016	-0.53 [-1.15; 0.09]
No 1998	0.10 [-0.67; 0.87]
Osborn 2010	0.10 [-0.48; 0.68]
Palmas 2014	-0.11 [-0.47; 0.25]
Perez-Escamilla 2015	-0.47 [-0.93; -0.01]
Philis-Tsimikas 2011	-0.10 [-0.72; 0.52]
Prezio 2013	-0.70 [-1.25; -0.15]
Ramal 2018	-2.18 [-3.18; -1.18]
Rosal 2005	-0.73 [-1.30; -0.16]
Rosal 2011	-0.53 [-0.92; -0.14]
Rothschild 2014	-0.55 [-0.95; -0.15]
Ruggiero 2014	0.25 [-2.61; 3.11]
Seligman 2018	0.24 [-0.09; 0.57]
Sixta 2008	0.13 [-0.44; 0.70]
Spencer 2018	-0.45 [-0.87; -0.03]
Sugiyama 2015	-0.40 [-0.75; -0.05]
Toobert 2011	-0.60 [-0.68; -0.52]
Vincent 2007	-0.70 [-1.61; 0.21]
Wagner 2016	0.20 [-0.48; 0.88]
Weinstock 2011	-0.33 [-0.55; -0.11]
Welch 2011	-1.00 [-1.80; -0.20]
Welch 2015	-0.80 [-1.08; -0.52]
TOTAL	-0.32 [-0.44; -0.20]

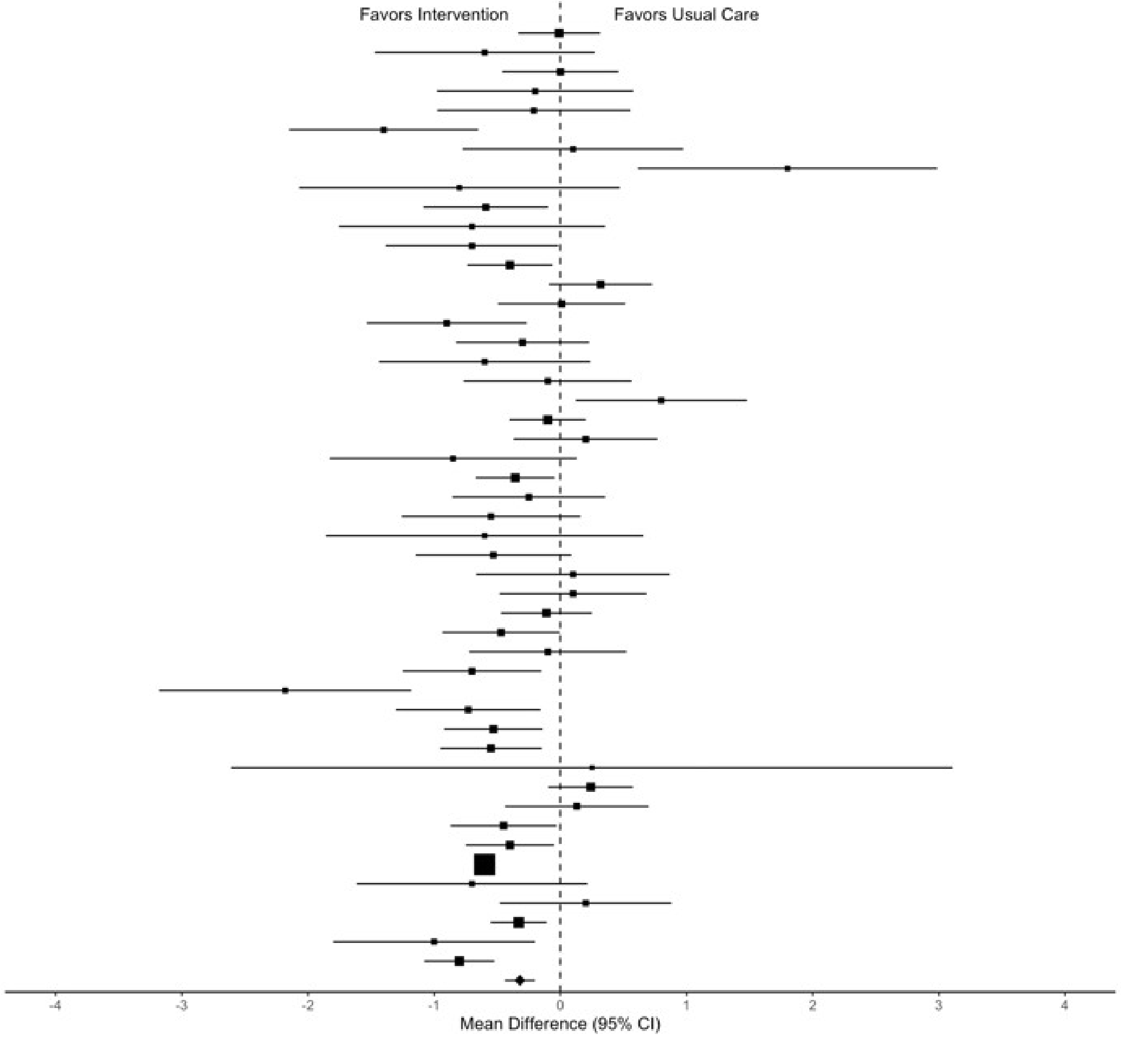

b. >50% participants prefer Spanish

Author	Mean Difference (95% CI)
Anderson 2010	-0.01 [-0.33; 0.31]
Aponte 2017	-0.60 [-1.47; 0.27]
Ayala 2015	0.00 [-0.46; 0.46]
Babamoto 2009	-0.20 [-0.98; 0.58]
Baig 2015	-0.21 [-0.97; 0.55]
Brown 2002	-1.40 [-2.15; -0.65]
Brown 2005	0.10 [-0.77; 0.97]
Brown 2011	1.80 [0.61; 2.99]
Burner 2018	-0.80 [-2.07; 0.47]
Carrasquillo 2017	-0.59 [-1.08; -0.10]
Castaneda 2004	-0.70 [-1.76; 0.36]
Castejon 2013	-0.70 [-1.38; -0.02]
Chamany 2015	-0.40 [-0.74; -0.06]
Ell 2011	0.01 [-0.49; 0.51]
Fortmann 2017	-0.90 [-1.53; -0.27]
Gerber 2005	-0.10 [-0.77; 0.57]
Heisler 2014	-0.10 [-0.40; 0.20]
Khanna 2014	0.20 [-0.37; 0.77]
Lorig 2008	-0.36 [-0.67; -0.05]
Lujan 2008	-0.25 [-0.86; 0.36]
McEwen 2017	-0.55 [-1.26; 0.16]
McKee 2011	-0.60 [-1.86; 0.66]
Osborn 2010	0.10 [-0.48; 0.68]
Perez-Escamilla 2015	-0.47 [-0.93; -0.01]
Philis-Tsimikas 2011	-0.10 [-0.72; 0.52]
Prezio 2013	-0.70 [-1.25; -0.15]
Ramal 2018	-2.18 [-3.18; -1.18]
Rosal 2005	-0.73 [-1.30; -0.16]
Rosal 2011	-0.53 [-0.92; -0.14]
Rothschild 2014	-0.55 [-0.95; -0.15]
Sixta 2008	0.13 [-0.44; 0.70]
Spencer 2018	-0.45 [-0.87; -0.03]
Vincent 2007	-0.70 [-1.61; 0.21]
Wagner 2016	0.20 [-0.48; 0.88]
Weinstock 2011	-0.33 [-0.55; -0.11]
TOTAL	-0.34 [-0.47; -0.21]

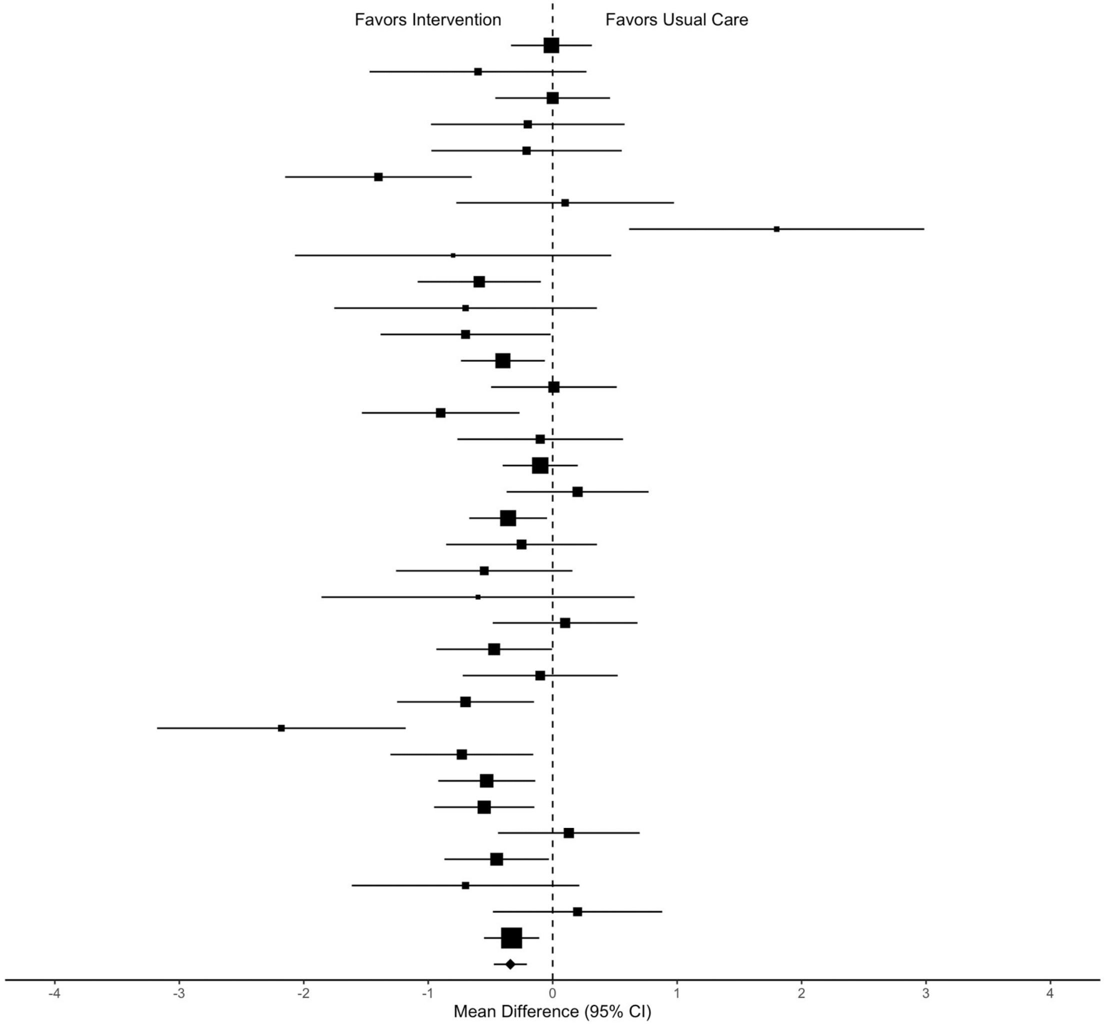

c. Single-level

Author	Mean Difference (95% CI)
Aponte 2017	-0.60 [-1.47; 0.27]
Carrasquillo 2017	-0.59 [-1.08; -0.10]
Castaneda 2004	-0.70 [-1.76; 0.36]
Castejon 2013	-0.70 [-1.38; -0.02]
Chamany 2015	-0.40 [-0.74; -0.06]
Christian 2008	0.32 [-0.09; 0.73]
Fortmann 2017	-0.90 [-1.53; -0.27]
Frosch 2011	-0.30 [-0.83; 0.23]
Gerber 2005a	-0.10 [-0.77; 0.57]
Gerber 2005b	0.80 [0.12; 1.48]
Heisler 2014	-0.10 [-0.40; 0.20]
Khanna 2014	0.20 [-0.37; 0.77]
Levy 2015	-0.85 [-1.83; 0.13]
Lujan 2008	-0.25 [-0.86; 0.36]
Moncrieft 2016	-0.53 [-1.15; 0.09]
No 1998	0.10 [-0.67; 0.87]
Osborn 2010	0.10 [-0.48; 0.68]
Palmas 2014	-0.11 [-0.47; 0.25]
Prezio 2013	-0.70 [-1.25; -0.15]
Rosal 2011	-0.53 [-0.92; -0.14]
Rothschild 2014	-0.55 [-0.95; -0.15]
Sixta 2008	0.13 [-0.44; 0.70]
Sugiyama 2015	-0.40 [-0.75; -0.05]
Wagner 2016	0.20 [-0.48; 0.88]
TOTAL	-0.25 [-0.40; -0.10]

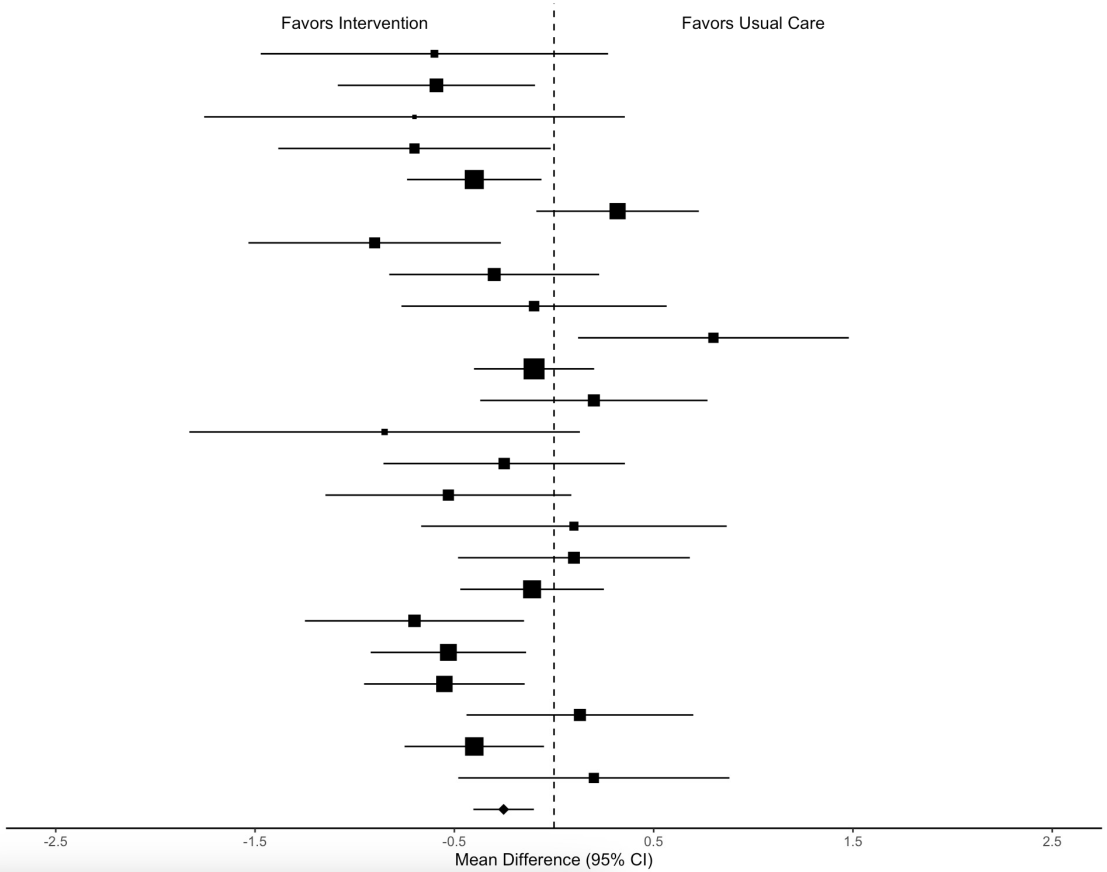

d. Single-domain

Author	Mean Difference (95% CI)
Aponte 2017	-0.60 [-1.47; 0.27]
Ayala 2015	0.00 [-0.46; 0.46]
Burner 2018	-0.80 [-2.07; 0.47]
Castaneda 2004	-0.70 [-1.76; 0.36]
Chamany 2015	-0.40 [-0.74; -0.06]
Frosch 2011	-0.30 [-0.83; 0.23]
Levy 2015	-0.85 [-1.83; 0.13]
Lorig 2008	-0.36 [-0.67; -0.05]
Moncrieft 2016	-0.53 [-1.15; 0.09]
No 1998	0.10 [-0.67; 0.87]
Palmas 2014	-0.11 [-0.47; 0.25]
Sugiyama 2015	-0.40 [-0.75; -0.05]
TOTAL	-0.32 [-0.46; -0.18]

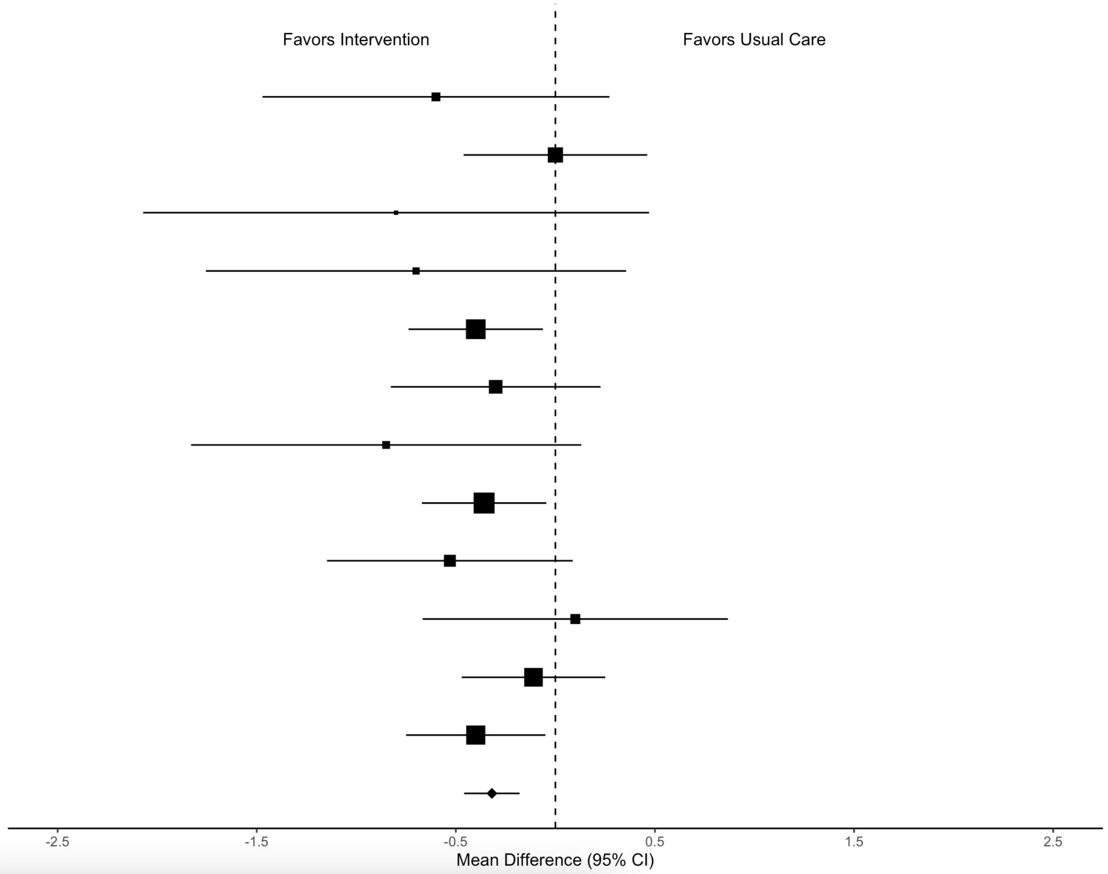

e. Single-level, single-domain

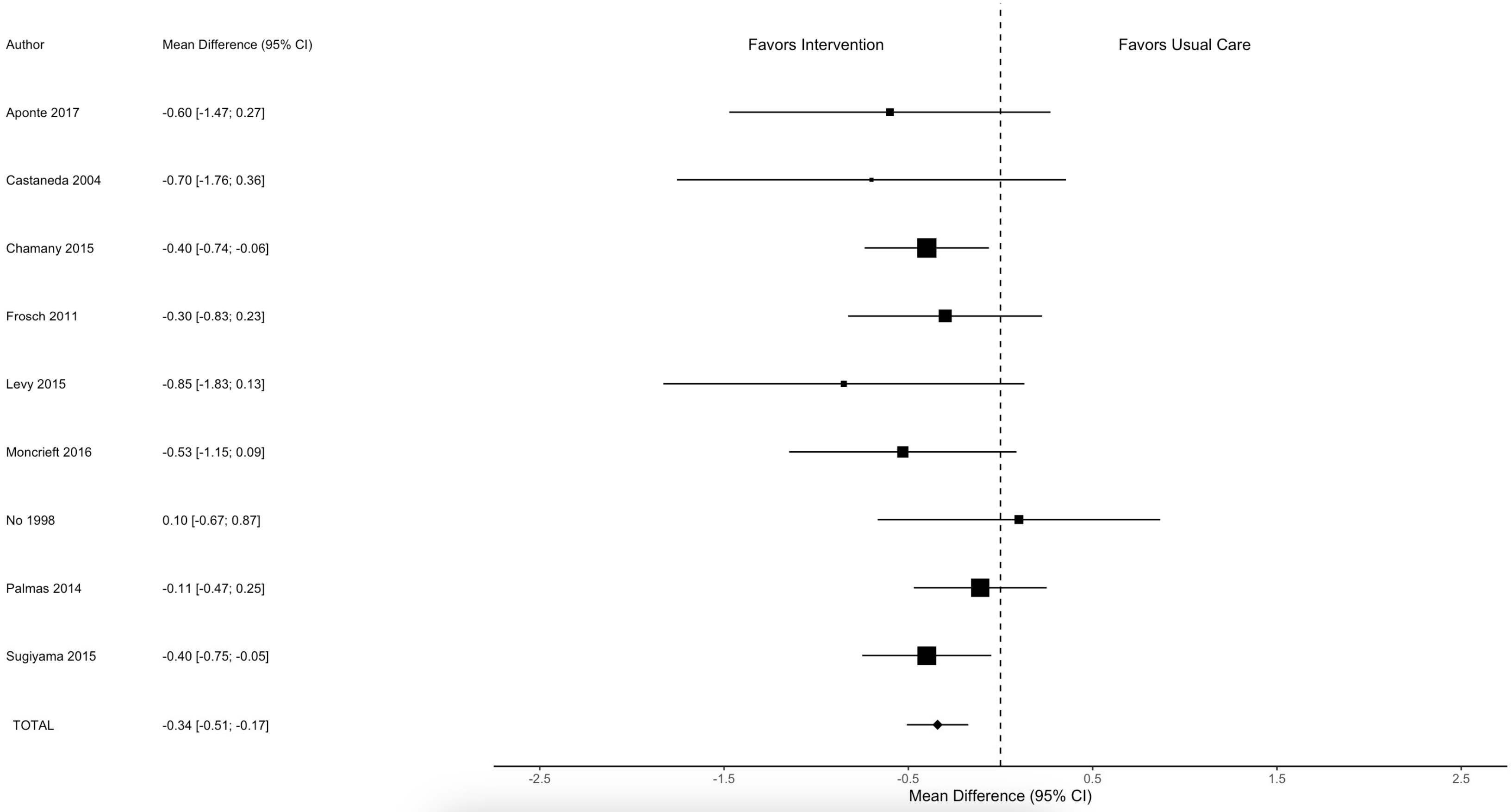

f. Multi-level

Author	Mean Difference (95% CI)
Anderson 2010	-0.01 [-0.33; 0.31]
Ayala 2015	0.00 [-0.46; 0.46]
Babamoto 2009	-0.20 [-0.98; 0.58]
Baig 2015	-0.21 [-0.97; 0.55]
Brown 2002	-1.40 [-2.15; -0.65]
Brown 2005	0.10 [-0.77; 0.97]
Brown 2011	1.80 [0.61; 2.99]
Burner 2018	-0.80 [-2.07; 0.47]
Ell 2011	0.01 [-0.49; 0.51]
Garcia 2014	-0.60 [-1.44; 0.24]
Lorig 2008	-0.36 [-0.67; -0.05]
McEwen 2017	-0.55 [-1.26; 0.16]
McKee 2011	-0.60 [-1.86; 0.66]
Perez-Escamilla 2015	-0.47 [-0.93; -0.01]
Philis-Tsimikas 2011	-0.10 [-0.72; 0.52]
Ramal 2018	-2.18 [-3.18; -1.18]
Rosal 2005	-0.73 [-1.30; -0.16]
Ruggiero 2014	0.25 [-2.61; 3.11]
Seligman 2018	0.24 [-0.09; 0.57]
Spencer 2018	-0.45 [-0.87; -0.03]
Toobert 2011	-0.60 [-0.68; -0.52]
Vincent 2007	-0.70 [-1.61; 0.21]
Weinstock 2011	-0.33 [-0.55; -0.11]
Welch 2011	-1.00 [-1.80; -0.20]
Welch 2015	-0.80 [-1.08; -0.52]
zTOTAL	-0.39 [-0.57; -0.21]

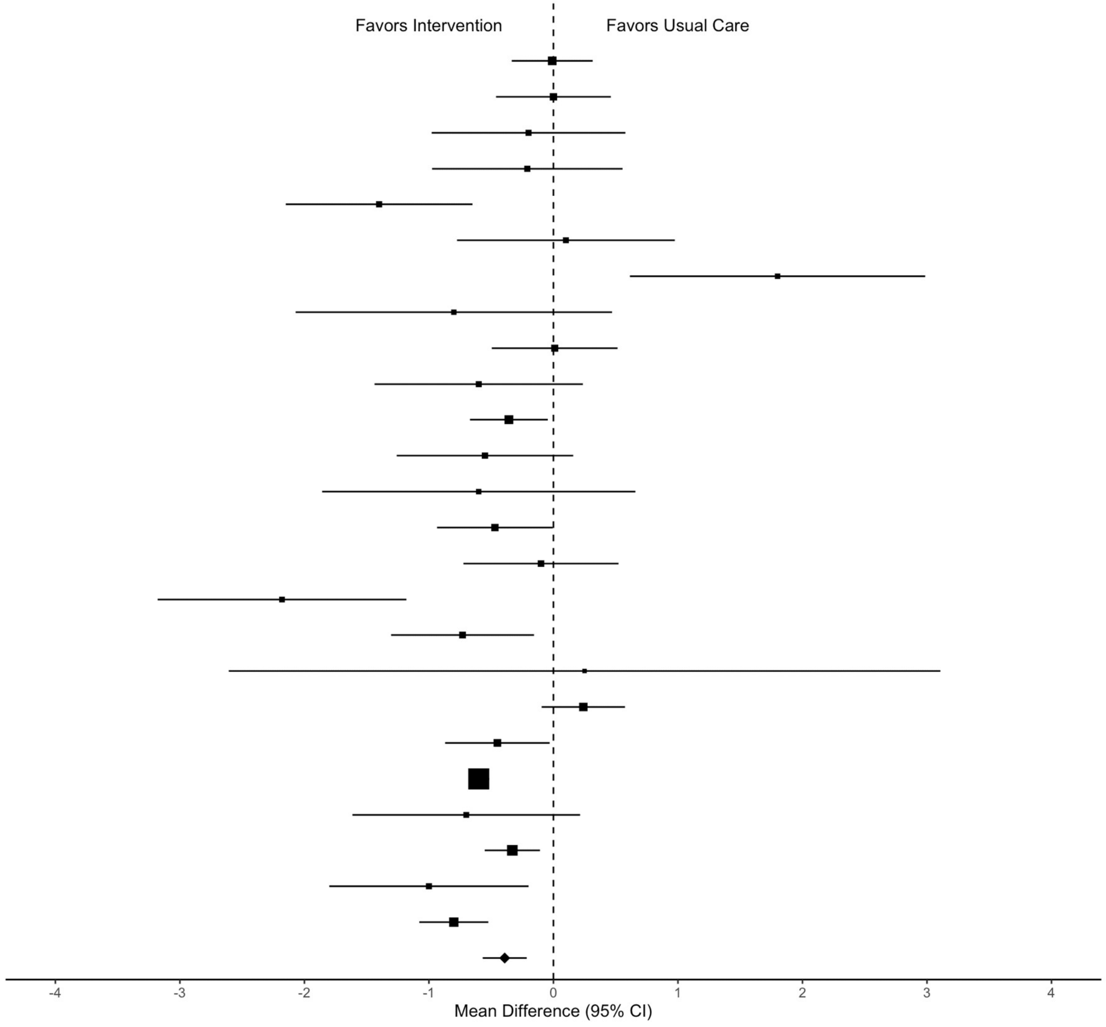

g. Multi-domain

Author	Mean Difference (95% CI)
Anderson 2010	-0.01 [-0.33; 0.31]
Babamoto 2009	-0.20 [-0.98; 0.58]
Baig 2015	-0.21 [-0.97; 0.55]
Brown 2002	-1.40 [-2.15; -0.65]
Brown 2005	0.10 [-0.77; 0.97]
Brown 2011	1.80 [0.61; 2.99]
Carrasquillo 2017	-0.59 [-1.08; -0.10]
Castejon 2013	-0.70 [-1.38; -0.02]
Christian 2008	0.32 [-0.09; 0.73]
Ell 2011	0.01 [-0.49; 0.51]
Fortmann 2017	-0.90 [-1.53; -0.27]
Garcia 2014	-0.60 [-1.44; 0.24]
Gerber 2005a	-0.10 [-0.77; 0.57]
Gerber 2005b	0.80 [0.12; 1.48]
Heisler 2014	-0.10 [-0.40; 0.20]
Khanna 2014	0.20 [-0.37; 0.77]
Lujan 2008	-0.25 [-0.86; 0.36]
McEwen 2017	-0.55 [-1.26; 0.16]
McKee 2011	-0.60 [-1.86; 0.66]
Osborn 2010	0.10 [-0.48; 0.68]
Perez-Escamilla 2015	-0.47 [-0.93; -0.01]
Philis-Tsimikas 2011	-0.10 [-0.72; 0.52]
Prezio 2013	-0.70 [-1.25; -0.15]
Ramal 2018	-2.18 [-3.18; -1.18]
Rosal 2005	-0.73 [-1.30; -0.16]
Rosal 2011	-0.53 [-0.92; -0.14]
Rothschild 2014	-0.55 [-0.95; -0.15]
Ruggiero 2014	0.25 [-2.61; 3.11]
Seligman 2018	0.24 [-0.09; 0.57]
Sixta 2008	0.13 [-0.44; 0.70]
Spencer 2018	-0.45 [-0.87; -0.03]
Toobert 2011	-0.60 [-0.68; -0.52]
Vincent 2007	-0.70 [-1.61; 0.21]
Wagner 2016	0.20 [-0.48; 0.88]
Weinstock 2011	-0.33 [-0.55; -0.11]
Welch 2011	-1.00 [-1.80; -0.20]
Welch 2015	-0.80 [-1.08; -0.52]
TOTAL	-0.31 [-0.46; -0.16]

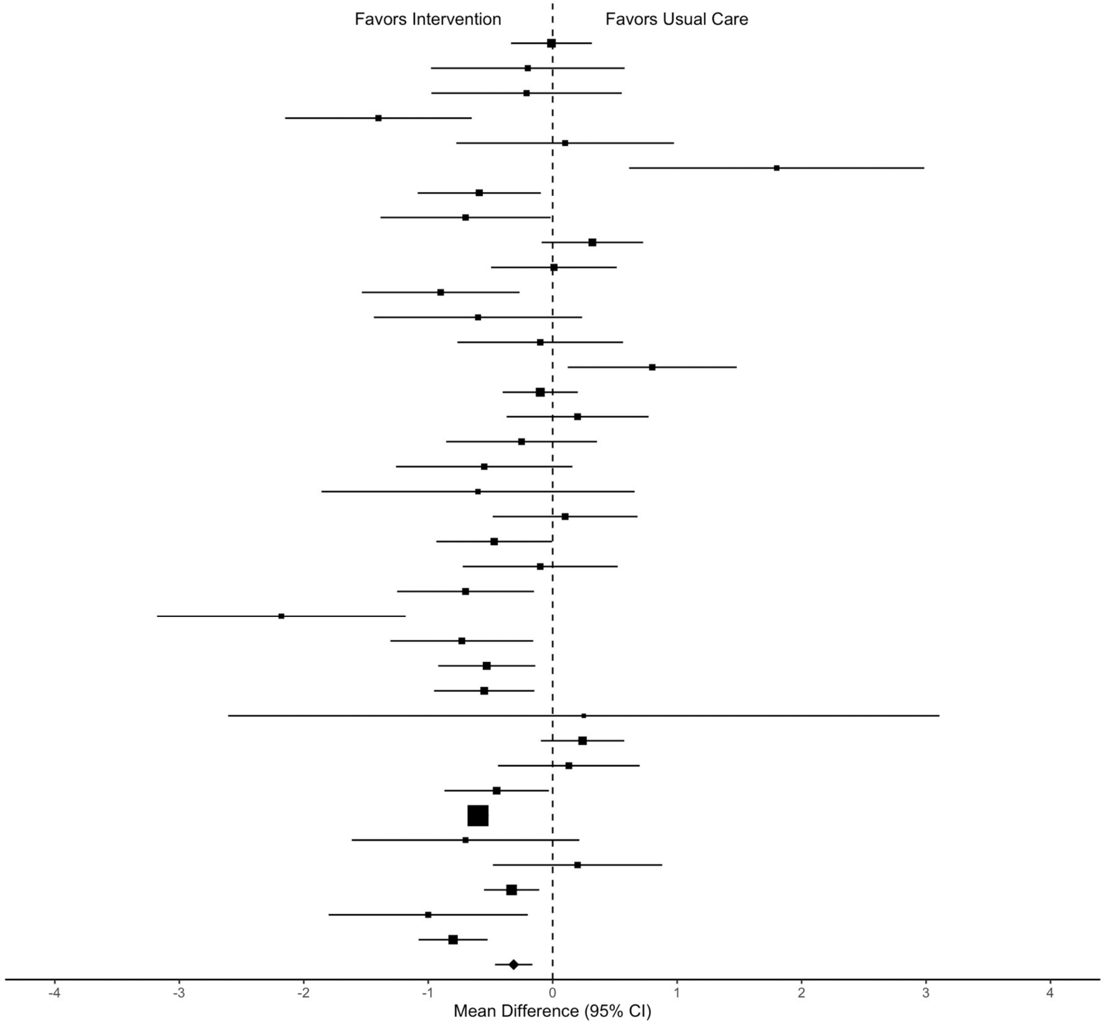

h. Multi-level, multi-domain

Author	Mean Difference (95% CI)
Anderson 2010	-0.01 [-0.33; 0.31]
Babamoto 2009	-0.20 [-0.98; 0.58]
Baig 2015	-0.21 [-0.97; 0.55]
Brown 2002	-1.40 [-2.15; -0.65]
Brown 2005	0.10 [-0.77; 0.97]
Brown 2011	1.80 [0.61; 2.99]
Ell 2011	0.01 [-0.49; 0.51]
Garcia 2014	-0.60 [-1.44; 0.24]
McEwen 2017	-0.55 [-1.26; 0.16]
McKee 2011	-0.60 [-1.86; 0.66]
Perez-Escamilla 2015	-0.47 [-0.93; -0.01]
Philis-Tsimikas 2011	-0.10 [-0.72; 0.52]
Ramal 2018	-2.18 [-3.18; -1.18]
Rosal 2005	-0.73 [-1.30; -0.16]
Ruggiero 2014	0.25 [-2.61; 3.11]
Seligman 2018	0.24 [-0.09; 0.57]
Spencer 2018	-0.45 [-0.87; -0.03]
Toobert 2011	-0.60 [-0.68; -0.52]
Vincent 2007	-0.70 [-1.61; 0.21]
Weinstock 2011	-0.33 [-0.55; -0.11]
Welch 2011	-1.00 [-1.80; -0.20]
Welch 2015	-0.80 [-1.08; -0.52]
TOTAL	-0.41 [-0.61; -0.21]

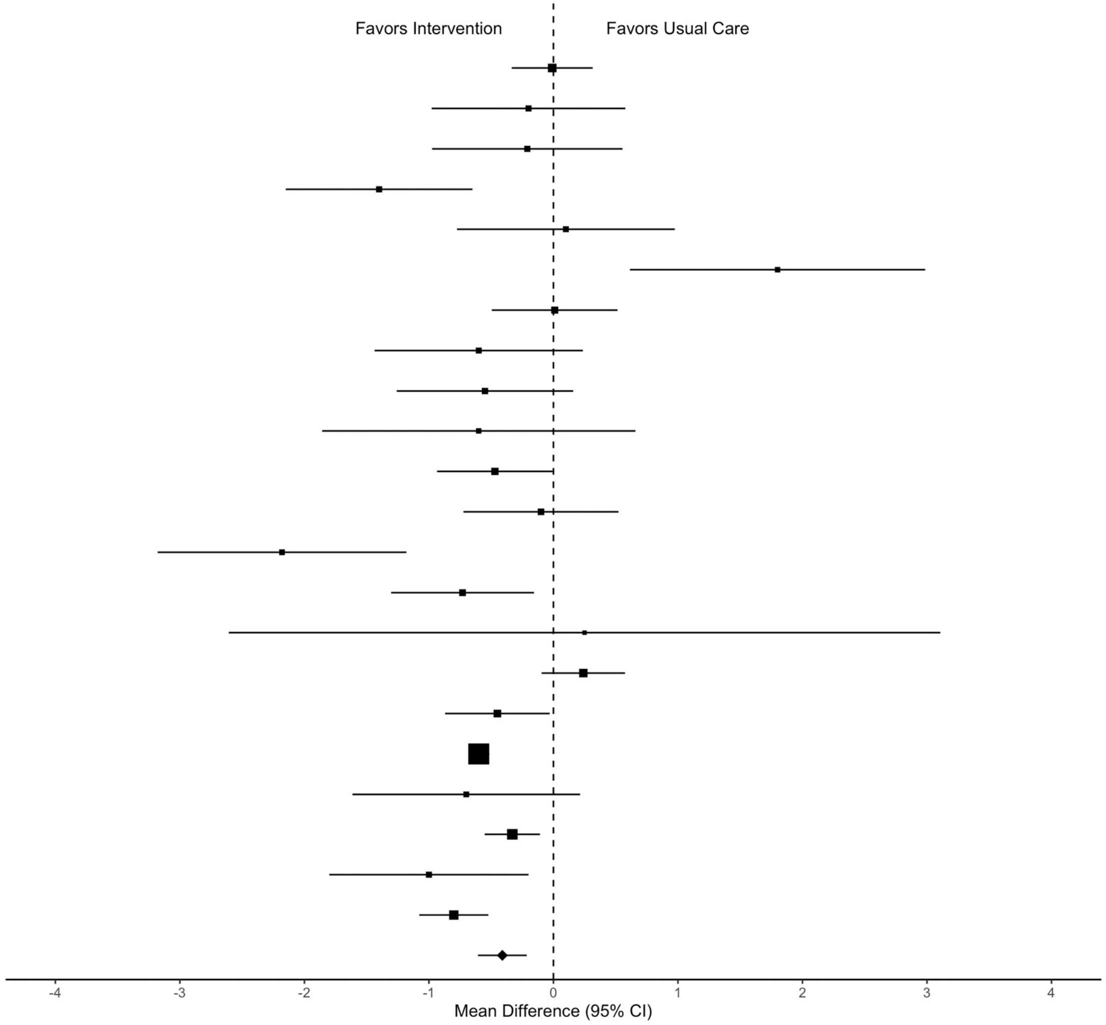

Supplemental Table S5. Risk of Bias in Randomized Controlled Trials of Non-Pharmacological Interventions Among U.S. Hispanic Populations with Type 2 Diabetes, 1985-2019

Study	Randomization Process	Deviations from Intended Intervention	Missing Outcome Data	Measurement of the Outcome	Selection of the Reported Results	Overall
Anderson 2010, [52]	low	low	low	low	low	low
Aponte 2017, [16]	low	low	low	low	low	low
Ayala 2015, [37]	low	low	low	low	low	low
Babamoto 2009, [53]	low	high	low	low	low	high
Baig 2015, [58]	low	low	some concerns	low	low	some concerns
Brown 2002, [40]	low	low	some concerns	low	low	some concerns
Brown 2005, [41]	low	low	low	low	low	low
Brown 2011, [42]	low	low	low	low	low	low
Burner 2018, [38]	low	low	low	low	low	low
Carrasquillo 2017, [24]	low	low	some concerns	low	low	some concerns
Castaneda 2002, [17]	low	low	low	low	low	low
Castejon 2013, [25]	low	low	some concerns	low	low	some concerns
Chamany 2015, [18]	low	low	low	low	low	low
Christian 2008, [61]	low	some concerns	low	low	low	some concerns
Ell 2011, [62]	low	low	some concerns	low	low	some concerns
Fortmann 2017, [26]	low	low	some concerns	low	low	some concerns
Frosch 2011, [19]	low	low	low	low	low	low
Garcia 2014, [59]	low	low	some concerns	low	low	some concerns
Gerber 2005, [27]	low	low	some concerns	low	low	some concerns
Heisler 2014, [28]	low	low	low	low	low	low
Khanna 2014, [29]	low	low	some concerns	low	low	some concerns
Levy 2015, [60]	low	low	low	low	low	low
Lorig 2008, [39]	low	some concerns	low	low	low	some concerns

Study	Randomization Process	Deviations from Intended Intervention	Missing Outcome Data	Measurement of the Outcome	Selection of the Reported Results	Overall
Lujan 2007, [30]	low	low	low	low	low	low
McEwen 2017, [43]	low	low	some concerns	low	low	some concerns
McKee 2011, [50]	low	low	low	low	low	low
Moncrieft 2016, [20]	low	low	low	low	low	low
Noel 1998, [21]	low	some concerns	low	low	low	some concerns
Osborn 2010, [31]	low	low	high	low	low	high
Palmas 2014, [22]	low	low	low	low	low	low
Philis-Tsimikas 2011, [44]	low	low	low	low	low	low
Prezio 2013, [32]	low	low	low	low	low	low
Perez-Escamilla 2015, [54]	low	low	low	low	low	low
Ramal 2018, [45]	low	low	low	low	low	low
Rosal 2005, [46]	low	low	low	low	low	low
Rosal 2011, [33]	low	low	some concerns	low	low	some concerns
Rothschild 2014, [34]	low	low	some concerns	low	low	some concerns
Ruggiero 2014, [55]	low	some concerns	some concerns	low	low	some concerns
Seligman 2018, [63]	low	low	low	low	low	low
Sixta 2008, [35]	low	some concerns	high	low	low	high
Spencer 2018, [47]	low	low	low	low	low	low
Sugiyama 2015, [23]	low	low	low	low	low	low
Toobert 2011, [48]	low	low	low	low	low	low
Vincent 2007, [49]	low	low	low	low	low	low
Wagner 2016, [36]	low	some concerns	low	low	low	some concerns
Weinstock 2011, [51]	low	some concerns	some concerns	low	low	some concerns
Welch 2011, [56]	low	some concerns	low	low	low	some concerns

Study	Randomization Process	Deviations from Intended Intervention	Missing Outcome Data	Measurement of the Outcome	Selection of the Reported Results	Overall
Welch 2015, [57]	low	low	low	low	low	low

Supplemental Figure S2. Funnel Plots

a. All trials
   Egger's test: p=0.03

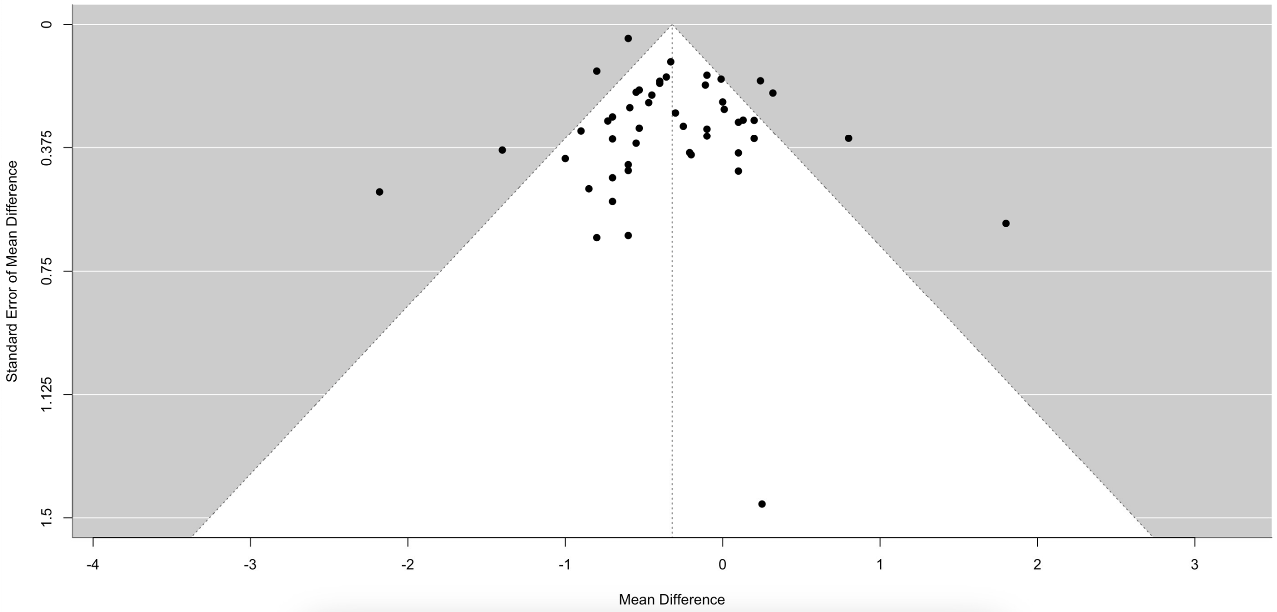

b. Trials in which >50% participants prefer Spanish
   Egger's test: p=0.06

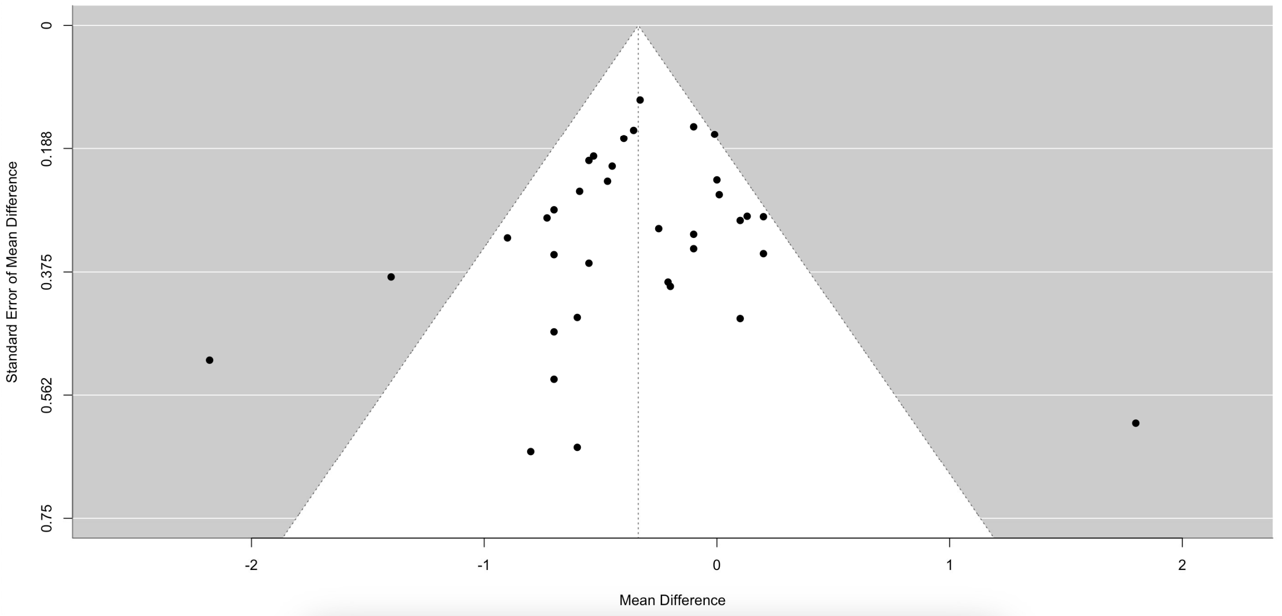
Standard Error of Mean Difference
0
0.188
0.375
0.562
0.75
-2
-1
0
1
2
Mean Difference

c. Multi-level interventions

Egger's test: p=0.06

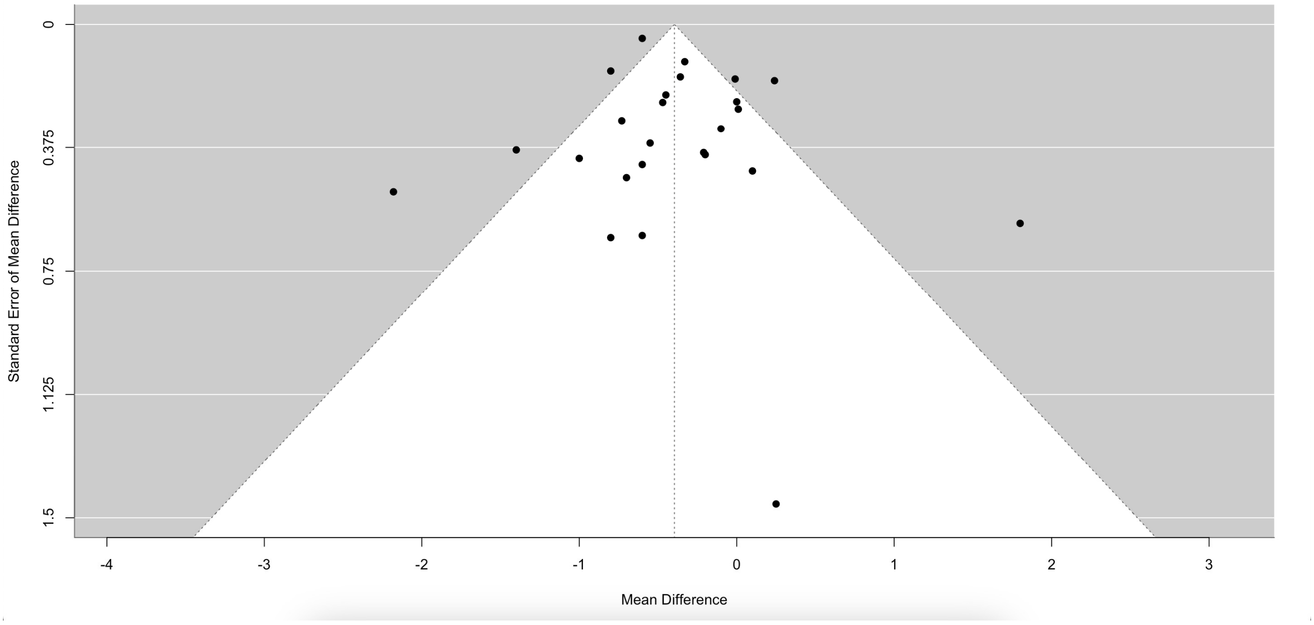

d. Multi-domain interventions

Egger's test: p=0.08

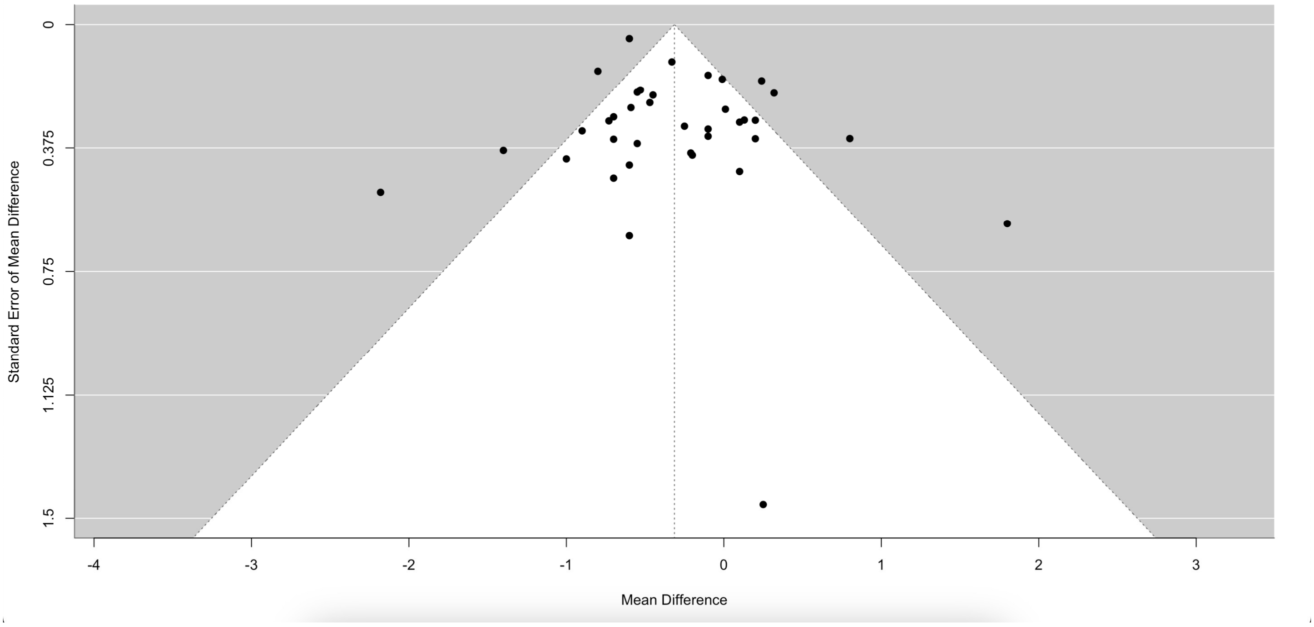

e. Multi-level, multi-domain interventions

Egger's test: p=0.09

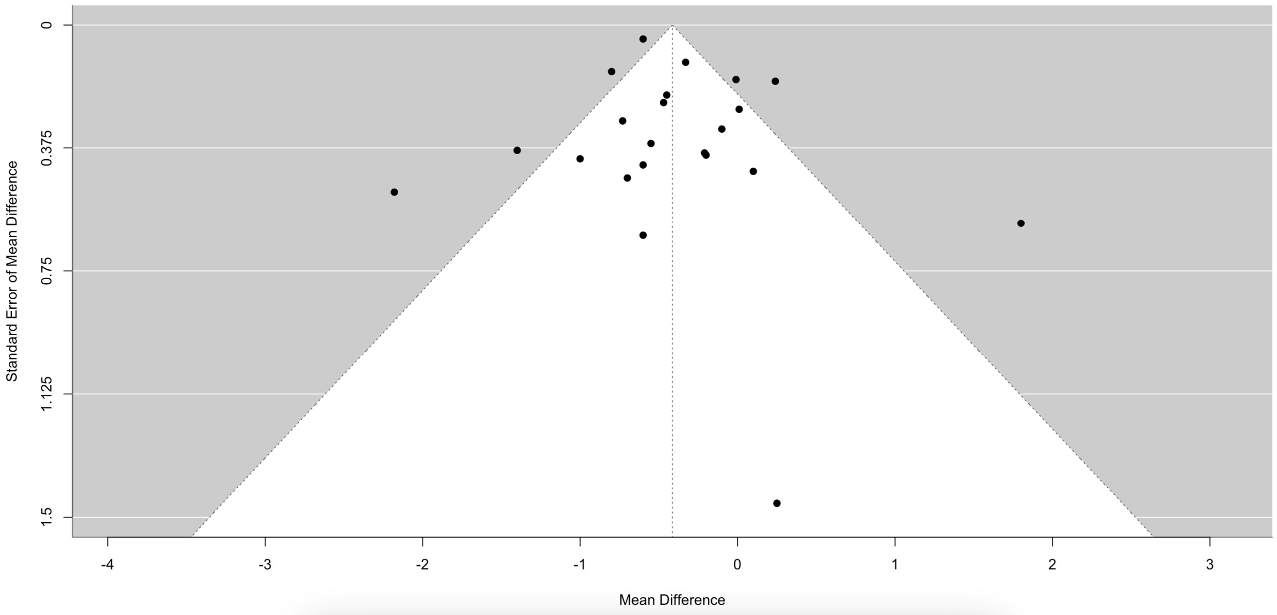

Supplemental Table S6. GRADE Strength of Evidence Assessment for Non-Pharmacological Interventions Among U.S. Hispanic Populations with Type 2 Diabetes

	N trials	Risk of Bias	Inconsistency	Indirectness	Imprecision	Publication Bias	Quality of Evidence
All trials	48	Low	No serious inconsistency	No serious indirectness	No serious imprecision	Possible bias detected	Moderate ■■■□
>50% participants prefer Spanish	35	Low	No serious inconsistency	No serious indirectness	No serious imprecision	Possible bias detected	Moderate ■■■□
Multi-level	25	Low	No serious inconsistency	Indirectness due to interventions*	No serious imprecision	Not detected	Moderate ■■■□
Multi-domain	36	Some concerns	Inconsistency	No serious indirectness	No serious imprecision	Not detected	Low ■■□□
Multi-level, multi-domain	22	Low	No serious inconsistency	Indirectness due to interventions*	No serious imprecision	Not detected	Moderate ■■■□

*Multi-level interventions included in meta-analysis focused on individual + interpersonal, cannot draw conclusions about multi-level in general

References for Included RCTs

1. Anderson, D. R.; Christison-Lagay, J.; Villagra, V.; Liu, H.; Dziura, J. Managing the space between visits: A randomized trial of disease management for diabetes in a community health center. *Journal of General Internal Medicine.* **2010** *25*(10), 1116–1122. https://doi.org/10.1007/s11606-010-1419-5
2. Aponte, J.; Jackson, T. D.; Wyka, K.; Ikechi, C.. Health effectiveness of community health workers as a diabetes self-management intervention. *Diabetes & Vascular Disease Research.* **2017** *14*(4), 316–326. https://doi.org/10.1177/1479164117696229
3. Ayala, G. X.; Ibarra, L.; Cherrington, A. L.; Parada, H.; Horton, L.; Ji, M.; Elder, J. P. Puentes hacia una mejor vida (Bridges to a Better Life): Outcome of a Diabetes Control Peer Support Intervention. *Annals of Family Medicine*, *13 Suppl 1*. **2015** S9-17. https://doi.org/10.1370/afm.1807
4. Babamoto, K. S.; Sey, K. A.; Camilleri, A. J.; Karlan, V. J.; Catalasan, J.; Morisky, D. E. Improving diabetes care and health measures among hispanics using community health workers: Results from a randomized controlled trial. *Health Education & Behavior: The Official Publication of the Society for Public Health Education.* **2009** *36*(1), 113–126. https://doi.org/10.1177/1090198108325911
5. Baig, A. A.; Benitez, A.; Locklin, C. A.; Gao, Y.; Lee, S. M.; Quinn, M. T.; Solomon, M. C.; Sánchez-Johnsen, L.; Burnet, D. L.; Chin, M. H.; Little Village Community Advisory Board. Picture Good Health: A Church-Based Self-Management Intervention Among Latino Adults with Diabetes. *Journal of General Internal Medicine.* **2015** *30*(10), 1481–1490. https://doi.org/10.1007/s11606-015-3339-x
6. Brown, S. A.; Blozis, S. A.; Kouzekanani, K.; Garcia, A. A.; Winchell, M.; Hanis, C. L. Dosage effects of diabetes self-management education for Mexican Americans: The Starr County Border Health Initiative. *Diabetes Care.* **2005** *28*(3), 527–532. https://doi.org/10.2337/diacare.28.3.527
7. Brown, S. A.; Garcia, A. A.; Kouzekanani, K.; Hanis, C. L. Culturally competent diabetes self-management education for Mexican Americans: The Starr County border health initiative. *Diabetes Care.* **2002** *25*(2), 259–268. https://doi.org/10.2337/diacare.25.2.259
8. Brown, S. A.; García, A. A.; Winter, M.; Silva, L.; Brown, A.; Hanis, C. L. Integrating education, group support, and case management for diabetic Hispanics. *Ethnicity & Disease.* **2011** *21*(1), 20–26.
9. Burner, E.; Lam, C. N.; DeRoss, R.; Kagawa-Singer, M.; Menchine, M.; Arora, S. Using Mobile Health to Improve Social Support for Low-Income Latino Patients with Diabetes: A Mixed-Methods Analysis of the Feasibility Trial of TExT-MED + FANS. *Diabetes Technology & Therapeutics.* **2018** *20*(1), 39–48. https://doi.org/10.1089/dia.2017.0198
10. Carrasquillo, O.; Lebron, C.; Alonzo, Y.; Li, H.; Chang, A.; Kenya, S. Effect of a Community Health Worker Intervention Among Latinos With Poorly Controlled Type 2 Diabetes: The Miami Healthy Heart Initiative Randomized Clinical Trial. *JAMA Internal Medicine.* **2017** *177*(7), 948–954. https://doi.org/10.1001/jamainternmed.2017.0926
11. Castaneda, C.; Layne, J. E.; Munoz-Orians, L.; Gordon, P. L.; Walsmith, J.; Foldvari, M.; Roubenoff, R.; Tucker, K. L.; Nelson, M. E. A randomized controlled trial of resistance exercise training to improve glycemic control in older adults with type 2 diabetes. *Diabetes Care*. **2002** *25*(12), 2335–2341. https://doi.org/10.2337/diacare.25.12.2335
12. Castejón, A. M., Calderón, J. L., Perez, A., Millar, C., McLaughlin-Middlekauff, J., Sangasubana, N., Alvarez, G., Arce, L., Hardigan, P., & Rabionet, S. E. A community-based pilot study of a diabetes pharmacist intervention in Latinos: Impact on weight and hemoglobin A1c. *Journal of Health Care for the Poor and Underserved*. **2013** *24*, 48–60. https://doi.org/10.1353/hpu.2014.0003
13. Chamany, S.; Walker, E. A.; Schechter, C. B.; Gonzalez, J. S.; Davis, N. J.; Ortega, F. M.; Carrasco, J.; Basch, C. E.; Silver, L. D. Telephone Intervention to Improve Diabetes Control: A Randomized Trial in the New York City A1c Registry. *American Journal of Preventive Medicine*. **2015** *49*(6), 832–841. https://doi.org/10.1016/j.amepre.2015.04.016
14. Christian, J. G.; Bessesen, D. H.; Byers, T. E.; Christian, K. K.; Goldstein, M. G.; Bock, B. C. Clinic-based support to help overweight patients with type 2 diabetes increase physical activity and lose weight. *Archives of Internal Medicine*. **2008** *168*(2), 141–146. https://doi.org/10.1001/archinternmed.2007.13
15. Ell, K.; Katon, W.; Xie, B.; Lee, P.-J.; Kapetanovic, S.; Guterman, J.; Chou, C.-P. One-year postcollaborative depression care trial outcomes among predominantly Hispanic diabetes safety net patients. *General Hospital Psychiatry*. **2011** *33*(5), 436–442. https://doi.org/10.1016/j.genhosppsych.2011.05.018
16. Fortmann, A. L.; Gallo, L. C.; Garcia, M. I.; Taleb, M.; Euyoque, J. A.; Clark, T.; Skidmore, J.; Ruiz, M.; Dharkar-Surber, S.; Schultz, J.; Philis-Tsimikas, A. Dulce Digital: An mHealth SMS-Based Intervention Improves Glycemic Control in Hispanics With Type 2 Diabetes. *Diabetes Care*. **2017** *40*(10), 1349–1355. https://doi.org/10.2337/dc17-0230
17. Frosch, D. L.; Uy, V.; Ochoa, S.; Mangione, C. M. Evaluation of a behavior support intervention for patients with poorly controlled diabetes. *Archives of Internal Medicine*. **2011** *171*(22), 2011–2017. https://doi.org/10.1001/archinternmed.2011.497
18. García, A. A.; Brown, S. A.; Horner, S. D.; Zuñiga, J.; Arheart, K. L. Home-based diabetes symptom self-management education for Mexican Americans with type 2 diabetes. *Health Education Research*. **2015** *30*(3), 484–496. https://doi.org/10.1093/her/cyv018
19. Gerber, B. S.; Brodsky, I. G.; Lawless, K. A.; Smolin, L. I.; Arozullah, A. M.; Smith, E. V.; Berbaum, M. L.; Heckerling, P. S.; Eiser, A. R. Implementation and evaluation of a low-literacy diabetes education computer multimedia application. *Diabetes Care*. **2005** *28*(7), 1574–1580. https://doi.org/10.2337/diacare.28.7.1574

20. Heisler, M.; Choi, H.; Palmisano, G.; Mase, R.; Richardson, C.; Fagerlin, A.; Montori, V. M.; Spencer, M.; An, L. C. Comparison of community health worker-led diabetes medication decision-making support for low-income Latino and African American adults with diabetes using e-health tools versus print materials: A randomized, controlled trial. *Annals of Internal Medicine*. **2014** *161*(10 Suppl), S13-22. https://doi.org/10.7326/M13-3012
21. Khanna, R.; Stoddard, P. J.; Gonzales, E. N.; Villagran-Flores, M.; Thomson, J.; Bayard, P.; Palos Lucio, A. G.; Schillinger, D.; Bertozzi, S.; Gonzales, R. An automated telephone nutrition support system for Spanish-speaking patients with diabetes. *Journal of Diabetes Science and Technology*. **2014** *8*(6), 1115–1120. https://doi.org/10.1177/1932296814550186
22. Levy, N.; Moynihan, V.; Nilo, A.; Singer, K.; Bernik, L. S.; Etiebet, M.-A.; Fang, Y.; Cho, J.; Natarajan, S. The Mobile Insulin Titration Intervention (MITI) for Insulin Adjustment in an Urban, Low-Income Population: Randomized Controlled Trial. *Journal of Medical Internet Research*. **2015** *17*(7), e180. https://doi.org/10.2196/jmir.4716
23. Lorig, K.; Ritter, P. L.; Villa, F.; Piette, J. D. Spanish diabetes self-management with and without automated telephone reinforcement: Two randomized trials. *Diabetes Care*. **2008** *31*(3), 408–414. https://doi.org/10.2337/dc07-1313
24. Lujan, J.; Ostwald, S. K.; Ortiz, M. Promotora diabetes intervention for Mexican Americans. *The Diabetes Educator*. **2007** *33*(4), 660–670. https://doi.org/10.1177/0145721707304080
25. McEwen, M. M.; Pasvogel, A.; Murdaugh, C.; Hepworth, J. Effects of a Family-based Diabetes Intervention on Behavioral and Biological Outcomes for Mexican American Adults. *The Diabetes Educator*. **2017** *43*(3), 272–285. https://doi.org/10.1177/0145721717706031
26. McKee, M. D.; Fletcher, J.; Sigal, I.; Giftos, J.; Schechter, C.; Walker, E. A. A collaborative approach to control hypertension in diabetes: Outcomes of a pilot intervention. *Journal of Primary Care & Community Health*. **2011** *2*(3), 148–152. https://doi.org/10.1177/2150131911401028
27. Moncrieft, A. E.; Llabre, M. M.; McCalla, J. R.; Gutt, M.; Mendez, A. J.; Gellman, M. D.; Goldberg, R. B.; Schneiderman, N. Effects of a Multicomponent Life-Style Intervention on Weight, Glycemic Control, Depressive Symptoms, and Renal Function in Low-Income, Minority Patients With Type 2 Diabetes: Results of the Community Approach to Lifestyle Modification for Diabetes Randomized Controlled Trial. *Psychosomatic Medicine*. **2016** *78*(7), 851–860. https://doi.org/10.1097/PSY.0000000000000348
28. Noël, P. H.; Larme, A. C.; Meyer, J.; Marsh, G.; Correa, A.; Pugh, J. A. Patient choice in diabetes education curriculum. Nutritional versus standard content for type 2 diabetes. *Diabetes Care*. **1998** *21*(6), 896–901. https://doi.org/10.2337/diacare.21.6.896
29. Osborn, C. Y.; Amico, K. R.; Cruz, N.; O'Connell, A. A.; Perez-Escamilla, R.; Kalichman, S. C.; Wolf, S. A.; Fisher, J. D. A brief culturally tailored intervention for Puerto Ricans with type 2 diabetes. *Health Education & Behavior: The Official Publication of the Society for Public Health Education*. **2010** *37*(6), 849–862. https://doi.org/10.1177/1090198110366004
30. Palmas, W.; Findley, S. E.; Mejia, M.; Batista, M.; Teresi, J.; Kong, J.; Silver, S.; Fleck, E. M.; Luchsinger, J. A.; Carrasquillo, O. Results of the northern Manhattan diabetes community outreach project: A randomized trial studying a community health worker intervention to improve diabetes care in Hispanic adults. *Diabetes Care*. **2014** *37*(4), 963–969. https://doi.org/10.2337/dc13-2142
31. Pérez-Escamilla, R.; Damio, G.; Chhabra, J.; Fernandez, M. L.; Segura-Pérez, S.; Vega-López, S.; Kollannor-Samuel, G.; Calle, M.; Shebl, F. M.; D'Agostino, D. Impact of a community health workers-led structured program on blood glucose control among latinos with type 2 diabetes: The DIALBEST trial. *Diabetes Care*. **2015** *38*(2), 197–205. https://doi.org/10.2337/dc14-0327
32. Philis-Tsimikas, A.; Fortmann, A.; Lleva-Ocana, L.; Walker, C.; Gallo, L. C. Peer-led diabetes education programs in high-risk Mexican Americans improve glycemic control compared with standard approaches: A Project Dulce promotora randomized trial. *Diabetes Care*. **2011** *34*(9), 1926–1931. https://doi.org/10.2337/dc10-2081
33. Prezio, E. A.; Cheng, D.; Balasubramanian, B. A.; Shuval, K.; Kendzor, D. E.; Culica, D. Community Diabetes Education (CoDE) for uninsured Mexican Americans: A randomized controlled trial of a culturally tailored diabetes education and management program led by a community health worker. *Diabetes Research and Clinical Practice*. **2013** *100*(1), 19–28. https://doi.org/10.1016/j.diabres.2013.01.027
34. Ramal, E.; Champlin, A.; Bahjri, K. Impact of a Plant-Based Diet and Support on Mitigating Type 2 Diabetes Mellitus in Latinos Living in Medically Underserved Areas. *American Journal of Health Promotion: AJHP*. **2018** *32*(3), 753–762. https://doi.org/10.1177/0890117117706793
35. Rosal, M. C.; Ockene, I. S.; Restrepo, A.; White, M. J.; Borg, A.; Olendzki, B.; Scavron, J.; Candib, L.; Welch, G.; Reed, G. Randomized trial of a literacy-sensitive, culturally tailored diabetes self-management intervention for low-income latinos: Latinos en control. *Diabetes Care*. **2011** *34*(4), 838–844. https://doi.org/10.2337/dc10-1981
36. Rosal, M. C.; Olendzki, B.; Reed, G. W.; Gumieniak, O.; Scavron, J.; Ockene, I. Diabetes self-management among low-income Spanish-speaking patients: A pilot study. *Annals of Behavioral Medicine: A Publication of the Society of Behavioral Medicine*. **2005** *29*(3), 225–235. https://doi.org/10.1207/s15324796abm2903_9
37. Rothschild, S. K.; Martin, M. A.; Swider, S. M.; Tumialán Lynas, C. M.; Janssen, I.; Avery, E. F.; Powell, L. H. Mexican American trial of community health workers: A randomized controlled trial of a community health worker intervention for Mexican Americans with type 2 diabetes mellitus. *American Journal of Public Health*. **2014** *104*(8), 1540–1548. https://doi.org/10.2105/AJPH.2013.301439

38. Ruggiero, L.; Riley, B. B.; Hernandez, R.; Quinn, L. T.; Gerber, B. S.; Castillo, A.; Day, J.; Ingram, D.; Wang, Y.; Butler, P. Medical assistant coaching to support diabetes self-care among low-income racial/ethnic minority populations: Randomized controlled trial. *Western Journal of Nursing Research*. **2014** *36*(9), 1052–1073. https://doi.org/10.1177/0193945914522862
39. Seligman, H. K.; Smith, M.; Rosenmoss, S.; Marshall, M. B.; Waxman, E. Comprehensive Diabetes Self-Management Support From Food Banks: A Randomized Controlled Trial. *American Journal of Public Health*. **2018** *108*(9), 1227–1234. https://doi.org/10.2105/AJPH.2018.304528
40. Sixta, C. S.; Ostwald, S. Texas-Mexico border intervention by promotores for patients with type 2 diabetes. *The Diabetes Educator*. **2008** *34*(2), 299–309. https://doi.org/10.1177/0145721708314490
41. Spencer, M. S.; Kieffer, E. C.; Sinco, B.; Piatt, G.; Palmisano, G.; Hawkins, J.; Lebron, A.; Espitia, N.; Tang, T.; Funnell, M.; Heisler, M. Outcomes at 18 Months From a Community Health Worker and Peer Leader Diabetes Self-Management Program for Latino Adults. *Diabetes Care*. **2018** *41*(7), 1414–1422. https://doi.org/10.2337/dc17-0978
42. Sugiyama, T.; Steers, W. N.; Wenger, N. S.; Duru, O. K.; Mangione, C. M. Effect of a community-based diabetes self-management empowerment program on mental health-related quality of life: A causal mediation analysis from a randomized controlled trial. *BMC Health Services Research*. **2015** *15*, 115. https://doi.org/10.1186/s12913-015-0779-2
43. Toobert, D. J.; Strycker, L. A.; King, D. K.; Barrera, M.; Osuna, D.; Glasgow, R. E. Long-term outcomes from a multiple-risk-factor diabetes trial for Latinas: ¡Viva Bien! *Translational Behavioral Medicine*. **2011** *1*(3), 416–426. https://doi.org/10.1007/s13142-010-0011-1
44. Vincent, D.; Pasvogel, A.; Barrera, L. A feasibility study of a culturally tailored diabetes intervention for Mexican Americans. *Biological Research for Nursing*. **2007** *9*(2), 130–141. https://doi.org/10.1177/1099800407304980
45. Wagner, J. A.; Bermudez-Millan, A.; Damio, G.; Segura-Perez, S.; Chhabra, J.; Vergara, C.; Feinn, R.; Perez-Escamilla, R. A randomized, controlled trial of a stress management intervention for Latinos with type 2 diabetes delivered by community health workers: Outcomes for psychological wellbeing, glycemic control, and cortisol. *Diabetes Research and Clinical Practice*. **2016** *120*, 162–170. https://doi.org/10.1016/j.diabres.2016.07.022
46. Weinstock, R. S.; Teresi, J. A.; Goland, R.; Izquierdo, R.; Palmas, W.; Eimicke, J. P.; Ebner, S.; Shea, S.; IDEATel Consortium. Glycemic control and health disparities in older ethnically diverse underserved adults with diabetes: Five-year results from the Informatics for Diabetes Education and Telemedicine (IDEATel) study. *Diabetes Care*. **2011** *34*(2), 274–279. https://doi.org/10.2337/dc10-1346
47. Welch, G.; Allen, N. A.; Zagarins, S. E.; Stamp, K. D.; Bursell, S.-E.; Kedziora, R. J. Comprehensive diabetes management program for poorly controlled Hispanic type 2 patients at a community health center. *The Diabetes Educator*. **2011** *37*(5), 680–688. https://doi.org/10.1177/0145721711416257
48. Welch, G.; Zagarins, S. E.; Santiago-Kelly, P.; Rodriguez, Z.; Bursell, S.-E.; Rosal, M. C.; Gabbay, R. A. (2015). An internet-based diabetes management platform improves team care and outcomes in an urban Latino population. *Diabetes Care*. **2015** *38*(4), 561–567. https://doi.org/10.2337/dc14-1412
